# Brain activation during processing of mouth actions in patients with disorders of consciousness

**DOI:** 10.1093/braincomms/fcae045

**Published:** 2024-02-15

**Authors:** Antonino Errante, Stefania Ferraro, Greta Demichelis, Chiara Pinardi, Mario Stanziano, Davide Sattin, Davide Rossi Sebastiano, Stefano Rozzi, Ludovico D’Incerti, Eleonora Catricalà, Matilde Leonardi, Maria Grazia Bruzzone, Leonardo Fogassi, Anna Nigri

**Affiliations:** Department of Medicine and Surgery, University of Parma, 43125 Parma, Italy; MOE Key Laboratory for Neuroinformation, School of Life Science and Technology, University of Electronic Science and Technology of China, 611731 Chengdu, China; Neuroradiology Unit, Diagnostic and Technology Department, Fondazione IRCCS Istituto Neurologico Carlo Besta, 20133 Milan, Italy; Neuroradiology Unit, Diagnostic and Technology Department, Fondazione IRCCS Istituto Neurologico Carlo Besta, 20133 Milan, Italy; Health Department, Fondazione IRCCS Istituto Neurologico Carlo Besta, 20133 Milan, Italy; Neuroradiology Unit, Diagnostic and Technology Department, Fondazione IRCCS Istituto Neurologico Carlo Besta, 20133 Milan, Italy; Neurosciences Department ‘Rita Levi Montalcini’, University of Turin, 10126 Turin, Italy; Istituti Clinici Scientifici Maugeri IRCCS, 20138 Milan, Italy; Neurophysiology Unit, Diagnostic and Technology Department, Fondazione IRCCS Istituto Neurologico Carlo Besta, 20133 Milan, Italy; Department of Medicine and Surgery, University of Parma, 43125 Parma, Italy; Neuroradiology Unit, Children’s Hospital A. Meyer—University of Florence, 50139 Florence, Italy; ICoN Cognitive Neuroscience Center, IUSS, Institute for Advances Studies, 27100 Pavia, Italy; Disability Unit and Coma Research Centre, Fondazione IRCCS Istituto Neurologico Carlo Besta, 20133 Milan, Italy; Neuroradiology Unit, Diagnostic and Technology Department, Fondazione IRCCS Istituto Neurologico Carlo Besta, 20133 Milan, Italy; Department of Medicine and Surgery, University of Parma, 43125 Parma, Italy; Neuroradiology Unit, Diagnostic and Technology Department, Fondazione IRCCS Istituto Neurologico Carlo Besta, 20133 Milan, Italy

**Keywords:** mirror neuron system, action observation, action verbs, disorders of consciousness, fMRI

## Abstract

In the past 2 decades, several attempts have been made to promote a correct diagnosis and possible restorative interventions in patients suffering from disorders of consciousness. Sensory stimulation has been proved to be useful in sustaining the level of arousal/awareness and to improve behavioural responsiveness with a significant effect on oro-motor functions. Recently, action observation has been proposed as a stimulation strategy in patients with disorders of consciousness, based on neurophysiological evidence that the motor cortex can be activated not only during action execution but also when actions are merely observed in the absence of motor output, or during listening to action sounds and speech. This mechanism is provided by the activity of mirror neurons. In the present study, a group of patients with disorders of consciousness (11 males, 4 females; median age: 55 years; age range: 19–74 years) underwent task-based functional MRI in which they had, in one condition, to observe and listen to the sound of mouth actions, and in another condition, to listen to verbs with motor or abstract content. In order to verify the presence of residual activation of the mirror neuron system, the brain activations of patients were compared with that of a group of healthy individuals (seven males, eight females; median age: 33.4 years; age range: 24–65 years) performing the same tasks. The results show that brain activations were lower in patients with disorders of consciousness compared with controls, except for primary auditory areas. During the audiovisual task, 5 out of 15 patients with disorders of consciousness showed only residual activation of low-level visual and auditory areas. Activation of high-level parieto-premotor areas was present in six patients. During the listening task, three patients showed only low-level activations, and six patients activated also high-level areas. Interestingly, in both tasks, one patient with a clinical diagnosis of vegetative state showed activations of high-level areas. Region of interest analysis on blood oxygen level dependent signal change in temporal, parietal and premotor cortex revealed a significant linear relation with the level of clinical functioning, assessed with coma recovery scale-revised. We propose a classification of the patient’s response based on the presence of low-level and high-level activations, combined with the patient’s functional level. These findings support the use of action observation and listening as possible stimulation strategies in patients with disorders of consciousness and highlight the relevance of combined methods based on functional assessment and brain imaging to provide more detailed neuroanatomical specificity about residual activated areas at both cortical and subcortical levels.

## Teaser

Errante *et al*. have studied the activation of the mirror neuron system (MNS) in patients with disorders of consciousness. Their findings demonstrate a spared activation of the MNS, thus suggesting the potential use of observation of actions and listening to their sound as stimulation programme in this challenging condition.

## Graphical Abstract

**Graphical Abstract:**
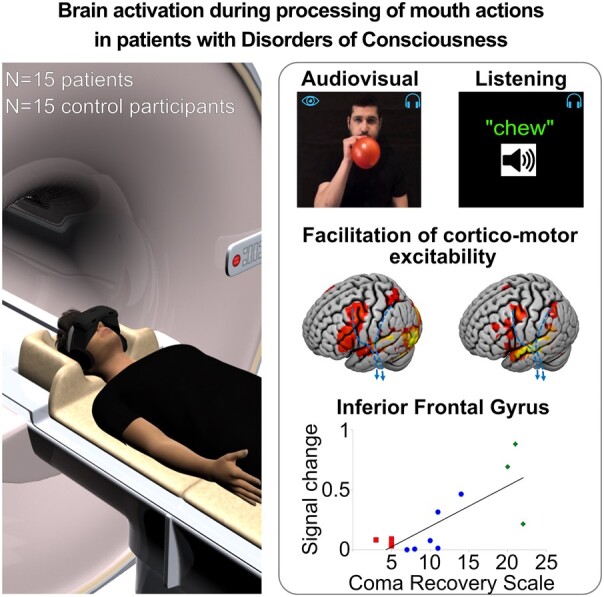

## Introduction

Patients surviving from a severe acute brain injury may encounter a condition of disorders of consciousness (DOC); after the initial acute comatose stage, characterized by no arousal and no awareness, these patients can evolve into the vegetative state (or unresponsive wakefulness syndrome—VS/UWS), characterized by a recovery of arousal and by no signs of awareness, or into the minimally conscious state (MCS), characterized by a recovery of arousal and minimal, reproducible but inconsistent signs of awareness.^1^

After the publication of the seminal study by Owen *et al*.,^2^ which showed that a patient classified as VS/UWS presented consistent neural activity related to an instructed imagery task—an activity incompatible with the diagnosis—it became clear that a fundamental issue is to make a correct diagnosis of these patients. In the diagnostic process, sensorimotor and language functions^3^ have been recognized as critical and dissociable factors with respect to the level of consciousness.^4^ Indeed, when either of these functions is impaired, it contributes to the underestimation of subtle signals of consciousness, hindering correct diagnosis and negatively affecting possible interactions with the external environment.

The knowledge gained in recent years allowed to achieve a more correct diagnostic framing in clinical practice: validated behavioural scales (e.g. coma recovery scale-revised—CRS-R and Motor Behaviour Tool-Revised) and new sophisticated diagnostic techniques^5-7^ are now limiting misdiagnoses^8,9^ and are enriching the DOC taxonomy. Patients with MCS are now stratified into MCS+ and MCS− based on the presence or absence of language expression and comprehension (i.e. responses to commands, intelligible verbalization, or yes/no responses), respectively,^10^ and the condition of cognitive-motor dissociation (CMD), characterized by volitional brain activity detected by task-based fMRI or EEG, unaccompanied by the corresponding volitional behaviour, is now identified as a new nosological entity.^4,11^ Remarkably, in this regard, neuroimaging data suggest that premotor–motor connectivity impairment and deterioration of the corticospinal tract characterize MCS and VS/UWS patients,^12^ while abnormalities in motor thalamo-cortical fibres characterize patients with CMD.^13^

Although the correct diagnosis and the different diagnostic categories are by no means a closed chapter, particularly in relation to the effects of language disorders, much research in recent years is now being devoted to identifying possible treatments aimed at recovering neural functions with the goal of supporting and promoting the recovery of consciousness. Currently, the only effective treatment in this direction in patients with severe traumatic brain injury is amantadine, a dopamine agonist and NMDA antagonist, as shown by a large class II randomized controlled trial.^14^ However, other few therapeutic options, mainly comprising brain stimulation and pharmacological and sensory stimulation treatments, still under investigation for their effects, are available (see for review Thibaut *et al*.^15^). Among these options, sensory stimulation programmes are the most widely employed.^16^ Several reasons underlie the wide application of these treatments: they have virtually no side effects, are simple to administer, do not require the supervision of trained technicians once established, can be easily transferred from specialized centres to home-based programmes and are relatively low cost.

Another possible stimulation strategy can be derived by Action Observation Treatment (AOT), already used for rehabilitation of several neurological disorders,^17,18^ that is based on the properties of the mirror neuron system (MNS). First discovered in monkey ventral premotor and inferior parietal cortex, mirror neurons (MNs) are a class of visuomotor neurons that activate not only during the execution of a motor act but also during observation of a similar motor act made by another agent.^19-21^ Subsequent studies employing brain imaging and electrophysiological techniques have demonstrated that the observation of motor acts activates a similar system in humans.^22,23^ This activation is somatotopically organized.^24^ Moreover, several studies reported the activation of the MNS in response to intransitive movements, including those without any obvious meaning. Evidence for this came mostly from TMS and fMRI techniques.^25-28^ Interestingly, it has also been demonstrated the existence of ‘audiovisual’ MNs, activated by the action sound in both monkeys^29^ and humans.^30^ In humans, also listening to action-related sentences (e.g. ‘grasp a glass’) elicits a somatotopic activation of the premotor cortex.^31^

On the basis of its properties, it has been hypothesized that the MNS plays a crucial role in imitation learning of new actions,^32^ thus providing the theoretical framework for using AOT to support motor rehabilitation in patients with stroke,^33,34^ Parkinson’s disease,^35,36^ aphasia^37^ and cerebral palsy.^38,39^ In fact, during the observation of an action performed by another agent, the MNS would recruit in the observer the same motor representations involved during the production of the observed action, facilitating corticomotor excitability, improving motor patterns^17^ and possibly supporting the partial recovery of cortico-peripheral motor pathways.^40^ Although a ‘real’ AOT is not feasible in patients with DOC since the imitative component might be absent, it is possible to hypothesize that also the simple observation of actions and listening to their sound might induce comparable neurophysiological and neuroplastic effects, thus facilitating the expression of intentional movements, in particular in patients with CMD.

Despite these possible positive effects, still to be proved in DOC patients, it is clear that support for the use of action observation/listening in DOC must be preceded by verification of the presence of MNS residual activity. Few previous studies tried to investigate the response of the MNS in DOC patients using task-based EEG techniques.^41-43^ In these studies, patients were required to perform a combined action observation/motor imagery task, or overt simple movement execution. Lechinger *et al*.,^43^ for example, investigated EEG oscillations in 10 VS/UWS and 7 MCS patients while they observed or imagined simple grasping motor acts, demonstrating that only MCS showed a clear spectral change in the theta, lower alpha and beta frequency band during both tasks. Interestingly, in their study, one patient clinically diagnosed as VS/UWS exhibited EEG patterns clearly resembling those of MCS patients.

Up to now, fMRI studies able to spatially localize residual MNS activity are not available. The aim of the present work was: (i) to verify, by using fMRI, the presence of residual MNS activity in patients with DOC or emerged from DOC, in order to provide a solid neurophysiological basis for the application of action observation/listening in these challenging conditions; (ii) to test whether the CRS-R scores, an index of the level of consciousness, is correlated with the presence of MNS activity; and (iii) to perform a classification of patients based on MNS activity, in an attempt to identify specific sub-categories that could benefit from action observation/listening. For this purpose, participants included in this study were administered with two different fMRI tasks: in one task, they had to observe and listen to the sound of goal-related mouth actions or intransitive movements, and in another task, to listen to verbs with motor or abstract content. We decided to use movies and sounds referred to oro-facial movements, starting from the common observation that rough voluntary and/or involuntary mouth motor patterns are relatively conserved in these patients. In addition, we used also intransitive movements to assess whether observation/listening of simple movements, as compared to goal-related actions, could effectively activate the MNS in DOC patients. The analyses of cortical activation focused both on low-level visual and auditory areas and on high-level areas, including the main nodes of the MNS. In addition, correlation analyses were carried out to verify the possible relation between MNS activation and patients’ global level of functioning.

## Materials and methods

### Patients and control participants

Fifteen patients with DOC and emerged from DOC (11 males, 4 females; median age: 55 years; age range: 19–74 years; median disease duration: 36 months; range: 7–120 months) (see Table 1) were consecutively recruited and assessed at the ‘Fondazione IRCCS Istituto Neurologico Carlo Besta’, Milan (IT). The sample included seven patients in VS/UWS, five in MCS and three with severe disability emerged from MCS (eMCS) characterized by different aetiologies, i.e. traumatic brain injury, haemorrhagic brain injury and anoxic brain injury. The demographic and clinical characteristics of the patients are reported in Table 1 and Supplementary Fig. 1. The main lesion localization is reported in Table 1.

**Table 1:** Summary of DOC patients recruited for the study, including demographic and clinical information

Patient ID	Age (years)	Sex	Aetiology	Time post-injury (months)	CRS-R score	CRS-R sub-scores: A-V-M-O-C-Ar	BAEP R/L	Main brain lesion localization
VS/UWS1^a^	19	M	TBI	50	6	1-0-2-1-0-2	NA	L cerebral hemisphere, brainstem Wallerian degeneration and atrophy
VS/UWS2	54	M	TBI	50	5	1-0-1-1-0-2	2/1	Bilateral cerebral axonal injury, brainstem Wallerian degeneration and atrophy
VS/UWS3^b^	58	F	ABI	21	7	1-1-2-1-0-2	1/1	Post-anoxic diffuse bilateral cortical atrophy, basal ganglia degeneration, brainstem atrophy
VS/UWS4	37	M	TBI	85	5	1-0-1-1-0-2	1/1	Bilateral (greater in the R hemisphere) cerebral axonal injury, brainstem Wallerian degeneration and atrophy
VS/UWS5^c^	62	F	ABI	76	8	2-1-2-1-0-2	1/0	Post-anoxic diffuse bilateral cortical atrophy, basal ganglia degeneration, brainstem atrophy
VS/UWS6	55	M	TBI	20	5	0-1-1-1-0-2	1/1	Bilateral cerebral (greater in the frontal lobes) axonal injury, brainstem Wallerian degeneration and atrophy
VS/UWS7	55	M	HBI	9	3	1-1-0-0-0-1	1/1	Post-haemorrhagic R fronto-parietal injury, diffuse cerebral atrophy
MCS-1	57	M	HBI	99	10	2-3-2-1-0-2	0/1	Post-haemorrhagic L temporo-parietal injury, brainstem Wallerian degeneration and atrophy
MCS-2	74	F	ABI	18	11	2-3-2-2-0-2	2/2	Post-anoxic diffuse bilateral cerebral atrophy (greater in the R hemisphere), superficial haemosiderosis, basal ganglia degeneration, brainstem atrophy
MCS-3	63	F	HBI	120	11	2-3-2-2-0-2	2/2	Post-haemorrhagic L cerebral hemisphere injury, diffuse cerebral atrophy, brainstem Wallerian degeneration and atrophy
MCS-4	51	M	HBI	8	10	1-3-3-1-0-2	NA	Post-haemorrhagic R fronto-temporal injury, superficial haemosiderosis, basal ganglia degeneration, brainstem atrophy
MCS+1	25	M	TBI	35	14	2-3-5-2-0-2	1/0	Bilateral cerebral (greater in the frontal lobes) axonal injury, brainstem Wallerian degeneration and atrophy
eMCS1	66	M	TBI	36	21	4-5-6-3-1-2	1/2	Post-traumatic L frontal lobe injury, R parietal subdural haematoma, brainstem Wallerian degeneration and atrophy
eMCS2	43	M	HBI	7	22	4-5-6-3-1-3	2/2	Post-haemorrhagic L thalamo-capsular injury
eMCS3	53	M	HBI	120	20	4-5-6-2-1-3	2/1	Post-haemorrhagic L corona radiata injury, L fronto-parietal atrophy, post-attinic T2* diffuse hypointensity

M, male; F, female; ABI, anoxic brain injury; HBI, haemorrhagic brain injury; TBI, traumatic brain injury; CRS-R, coma recovery scale-revised; A, auditory subscale score; V, visual subscale score; M, motor subscale score; O, oromotor subscale score; C, communication subscale score; Ar, arousal subscale score.

^a^No significant activation in AV/LS tasks.

^b^No significant activation in AV task.

^c^No significant activation in LS task.

In order to evaluate the brain activations evoked by the functional tasks, 15 healthy participants were recruited (7 males; median age: 33.4 years; age range: 24–65 years). All healthy participants had a normal or corrected-to-normal vision, no history of neurological, orthopaedic or rheumatological disorders. They were right-handed according to the Edinburgh Handedness Inventory,^44^ and matched by age with DOC patients (Mann–Whitney U = 69; *P* = 0.08). All legally authorized representatives of the patients and healthy participants provided written informed consent according to the Declaration of Helsinki prior to their inclusion in the study. This study was approved by the Fondazione IRCCS ‘Istituto Neurologico C. Besta’ Ethics Committee (Protocol 50/2018 of 11 April 2018).

### Clinical and neurophysiological assessment

All patients underwent multimodal assessment, including clinical, neurophysiological and neuroradiological evaluations. Patients were assessed using the Italian version of CRS-R.^45,46^ The MCS patients were subcategorized into MCS− and MCS+.^10^ All patients underwent a brainstem auditory-evoked potential (BAEP) test assessing the auditory-evoked potentials. BAEPs were evaluated as absent, altered or normal according to Nigri *et al*.^47^ as reported in Table 1.

### MRI acquisition and setup

All participants were scanned with a 3 T magnetic resonance imaging (MRI) system (Achieva, Philips Healthcare BV, Best, NL) using a 32-channel head coil. See Supplementary material for details about the employed MRI sequences. Stimuli were presented by means of a digital goggles system (Resonance Technology, Northridge, CA) (60 Hz refresh rate) with a resolution of 800 horizontal pixels × 600 vertical pixels and horizontal eye field of 30°. Auditory stimuli were presented by MR-compatible headphones. Software E-Prime 2 Professional (Psychology Software Tools, Inc.; http://www.pstnet.com) was used for stimuli presentation. For all participants, the sound volume was set to a comfortable level (from 85–90 dB) within the scanner such that stimuli were clearly audible and distinguishable.

#### fMRI experimental design

**Figure 1:**
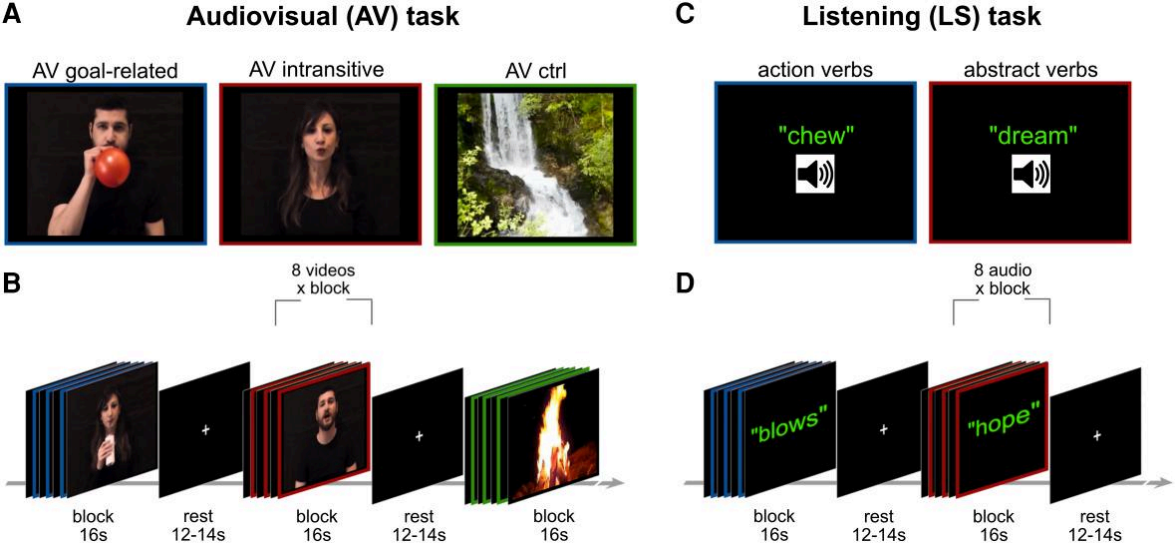
**Experimental design, stimuli and tasks.** (**A**) Static frames taken from the video stimuli used for the audiovisual (AV) task. Each frame shows a representative scene taken from the three categories of presented videos, i.e. mouth goal-related actions, mouth intransitive movements and environmental natural landscapes. (**B**) AV paradigm, made by independent blocks, each composed by eight videos of the same condition, interspersed with the rest condition. (**C**) Classes of the auditory stimuli administered during the listening (LS) task, including action verbs and abstract verbs. (**D**) LS auditory paradigm made by independent blocks, each composed by eight verbs of the same condition, interspersed with rest. ctrl, control condition.

During a single MRI scan, the participants performed two tasks: (i) an audiovisual (AV) task in which they observed goal-related and intransitive mouth actions, and heard the sound produced by these actions, or, as control, observed environmental landscapes and heard their noise; and (ii) a listening (LS) task in which the participants listened to mouth-action-related verbs, and, as control, to abstract verbs (Fig. 1). Two consecutive fMRI runs for each task were acquired. The presentation order of the AV and LS tasks was balanced across participants.

#### Audiovisual task stimuli

Audiovisual stimuli consisted of videos (see Fig. 1A) showing goal-related mouth actions (AV goal-related) or intransitive mouth movements (AV intransitive), performed by two actors (male, female) with the mouth, from a frontal perspective. All videos were recorded by means of a digital HD camera (© GoPro, Inc., USA), with a frame rate of 100/s and resolution of 1280 × 720p. Stimuli were resized and edited using Final Cut Pro X (v 10.5.1, Apple Inc.). They measured 14.7° by 14.7° and lasted 2 s. The objects used in the goal-related mouth action videos were an apple, a balloon and a straw. Four types of action were presented: ‘bite an apple’, ‘drink from a straw’, ‘inflate a balloon’ and ‘blow into a straw’. The AV intransitive videos represented four types of mouth intransitive movements: ‘to kiss’, ‘to snap the tongue’, ‘to whistle’ and ‘to spit’. A total of 12 video clips were recorded (2 actors × 2 conditions × 4 actions/movement types). As a control condition, an AV version of naturalistic scenes including ‘waterfall’, ‘sea in backwash’, ‘thunderstorm’ and ‘wind in the tree’ was presented, in order to investigate the brain activations evoked by the observation/listening of videos without motor content (AV ctrl).

#### Listening task stimuli

Audio stimuli consisted of Italian action verbs (https://www.ilc.cnr.it/risorse/) lasting 2 s. More specifically, four different mouth action verbs and four abstract verbs were presented, in the imperative mood [Italian used verbs: ‘sputa’ (spit), ‘soffia’ (blow), ‘mastica’ (chew), ‘bacia’ (kiss), ‘medita’ (meditate), ‘sogna’ (dream), ‘ignora’ (ignore), ‘dubita’ (doubt)]. Linguistic properties of the employed verbs are reported in Supplementary Table 1. All audio stimuli were pronounced by a native Italian actor and a native Italian actress using a cardioid condenser microphone (RODE NT1) placed 30 cm from the speaker and digitized with an A/D converter module with phantom power supply (M-AUDIO M-TRACK). The audio stimuli were then edited with FL Studio 11 software.

#### Audiovisual task procedure

The AV task was acquired using a block paradigm (Fig. 1B). Each block lasted 16 s, and it was composed of eight consecutive videos of the same condition. During each AV run, a total of 12 blocks of stimuli were presented, 4 blocks for each condition. Each goal-related action, intransitive movement and control video was presented 32 times per run. The order of blocks was counterbalanced across subjects. Blocks of stimuli were interleaved by a rest condition, used as baseline, lasting 12 or 14 s, in which a white cross was presented in the middle of a black screen. The duration of each AV run was ∼6 min.

#### Listening task procedure

In the LS task, participants were asked to attentively listen to the auditory stimuli (Fig. 1C), presented in blocks of eight stimuli of the same condition (action verbs, abstract verbs) (Fig. 1D). During each LS run, a total of 16 blocks of stimuli were presented, 8 blocks for each condition. Each condition (action verbs, abstract verbs) was presented 64 times per run. The order of blocks was counterbalanced across subjects and interleaved by the rest condition, used as the baseline, lasting 12 or 14 s. Each LS task run lasted ∼7 min.

### Statistical analysis

#### fMRI data preprocessing

See Supplementary material for a complete description of the MRI data preprocessing pipeline.

##### Single-subject whole-brain fMRI analysis

The pre-processed functional data for each participant were entered in single-subject whole-brain analysis.^48^ The single-subject fMRI responses were modelled using two different General Linear Models (GLMs). The first GLM (AV task) included four predictors (‘AV goal-related’, ‘AV intransitive’, ‘AV ctrl’, ‘baseline’), while the second GLM (LS task) included three predictors (‘LS action verbs’, ‘LS abstract verbs’, ‘baseline’). All regressors were composed of eight consecutive stimuli (events), which were modelled as one single epoch lasting 16 s. Both GLMs included six predictors of no interest obtained from the realignment process. We assessed blood oxygen level dependent (BOLD) signal changes using *t* statistical parametric maps in single participants with the following contrasts:

- ‘AV goal-related effect’, comparing the activation evoked by mouth goal-related actions with baseline;
- ‘AV intransitive effect’, comparing the activation during the processing of mouth intransitive movements with baseline;
- ‘AV ctrl effect’, comparing the activation during the processing of control stimuli with baseline;
- ‘LS action verbs effect’, comparing the activation during listening of action verbs with baseline; and
- ‘LS abstract verbs effect’, comparing the activation associated with listening of abstract verbs with baseline.

We showed single-subject whole-brain activity maps applying a statistical threshold of *P* < 0.001 corrected at cluster level for multiple comparisons using the false discovery rate (FDR) procedure and a minimum cluster size of five voxels.

##### fMRI whole-brain group analysis in healthy participants

Because of the high degree of variability in brain responses observed in patients, we performed a second-level, group-based statistical analysis only on healthy participants. The corresponding *t*-contrast maps derived from the first-level models were entered in two flexible ANOVAs with sphericity correction for repeated measures.^49^ A second-level model (AV task) included three regressors (‘AV goal-related effect’, ‘AV intransitive effect’, ‘AV ctrl effect’). Within this model, we also assessed the activations resulting from the direct contrasts between ‘AV goal-related’ versus ‘AV ctrl’ and ‘AV intransitive’ versus ‘AV ctrl’. Inclusive masks derived from the corresponding contrasts ‘AV goal-related’ versus ‘baseline’ and ‘AV intransitive’ versus ‘baseline’ were used (*P* < 0.05). This analysis aimed at identifying the brain areas involved in the processing of the observed actions, controlling for the effect of basic visual and auditory elaboration. This contrast also reveals the parietal and frontal high-level areas belonging to the MNS.

The remaining second-level model (LS task) included two regressors (‘LS action verbs effect’ and ‘LS abstract verbs effect’). Within this model, we also computed the activation map resulting from the direct contrast between ‘LS action verbs’ versus ‘LS abstract verbs’, inclusive mask LS ‘action verbs’ versus ‘baseline’ (*P* < 0.05). This analysis aimed at identifying the high-level brain areas engaged during the listening of verbs with motor content while controlling for basic auditory and speech processing. Furthermore, this contrast is aimed at localizing MNS areas activated by action verbs.

Statistical inference was drawn at the cluster level, with a threshold of *P* < 0.001 corrected for multiple comparisons using FDR correction and a minimum cluster size of five voxels. Activation peaks were localized with reference to cytoarchitectonic probabilistic maps of the human brain, using the SPM-Anatomy toolbox v3.0.^50^

##### Identification of regions of interest

To test the relationship between the MNS activity and patients’ diagnosis and to perform a classification of patients based on MNS activity, we employed a region of interest (ROI)-based approach. Starting from the results obtained in second-level analyses in healthy participants, we identified a set of cortical areas activated during the AV and LS tasks. Local maxima of significant clusters activated during the audiovisual and/or the listening tasks were identified, as reported in Supplementary Fig. 2 (statistical threshold set at *P* < 0.001, FDR corrected at cluster level). Regions defined based on local maxima included: (i) low-level visual and auditory areas involved in the processing of basic features of the stimuli; and (ii) high-level cortical ROIs, including premotor and parietal areas belonging to the MNS. We created ROI masks to extract BOLD signal change using an anatomical approach, instead of the specific activation coordinates, to avoid any circularity issue in ROIs localization. In particular, ROI masks were selected from the AAL atlas,^51^ the Wake Forest University PickAtlas^52^ (WFU PickAtlas) and the Human Motor Area Template^53^ (HMAT). The following low-level visual and auditory ROIs were included: (i) left/right primary visual area (V1); (ii) left/right superior temporal gyrus (STG), including the primary auditory cortex; and (iii) left/right MT/V5 area. The high-level cortical ROIs included: (i) left/right intraparietal sulcus (IPS); (ii) left/right supramarginal gyrus (SMG), including the ventral sector of the SMG and the rostral most section of the central angular gyrus; (iii) left/right ventral sector of the precentral gyrus (PMv); (iv) left/right dorsal sector of the precentral gyrus (PMd); (v) left/right inferior frontal gyrus, pars opercularis (IFGop); and (vi) left/right inferior frontal gyrus pars triangularis (IFGtria).

##### Relation between ROIs activations and diagnosis

For each patient, we extracted in each ROI the average BOLD signal change across all significant voxels using the SPM Rex Toolbox (http://web.mit.edu/swg/rex). Then BOLD signal changes of relevant AV contrasts (i.e. ‘AV goal-related effect’ and ‘AV intransitive effect’) and LS contrasts (i.e. ‘LS action verbs effect’ and ‘LS abstract verbs effect’) were averaged and used in regression analyses testing for a linear relation between brain activity during AV/LS tasks and CRS-R score, in the ROIs selected for the analysis. The normal distribution of variables was assessed by the Shapiro–Wilk test.

##### Patients’ classification based on functional brain activity

To test if the presence/degree of brain activation in low-level and high-level ROIs belonging to the action observation/listening network significantly predicted the patient’s functioning, we classified the patients, for each task, into three subgroups: (i) patients showing no significant activity in any ROI (score = 0); (ii) patients showing significant activity only in low-level visual and auditory ROIs (score = 1); and (iii) patients showing significant activity in both low-level auditory and visual ROIs and high-level cortical ROIs (score = 2). For the AV task, we considered the activity in the ROI during ‘AV goal-related effect’ and ‘AV intransitive effect’, while for the LS task for both ‘LS action verbs effect’ and ‘LS abstract verbs effect’. Then, we carried out linear regression analysis entering as variables the CRS-R scores and the scores of the brain functional response.

##### Lesion topography

To quantify the structural alterations, two expert neuroradiologists (L.D. and M.S.) evaluated the severity of gross anatomical and signal abnormality of cortical and subcortical ROIs in both hemispheres, blinded to patients’ diagnosis, using the following qualitative scale: score 0 (severely damaged, i.e. parenchyma obliterated and/or intense, pervasive hyperintensity), score 1 (recognizable but distorted morphology and/or severe signal abnormality), score 2 (moderate anatomical damage and/or signal abnormality), score 3 (mild anatomical damage and/or signal abnormality) and score 4 (normal appearance).^54^ Structural abnormalities were identified by means of MRI morphological scans (i.e. T1w and Fluid Attenuated Inversion Recovery - FLAIR). This approach outperformed the quantitative one, which resulted to be hardly feasible in chronic DOC patients.^55^ The ROIs assessed include the low-level and high-level cortical ROIs (see ‘Identification of regions of interest’), plus a set of subcortical ROIs from the AAL atlas known to be crucial for the maintenance of a residual level of consciousness (i.e. midbrain and thalamus) and for the execution of correct voluntary movements (i.e. left/right putamen, caudate nucleus and globus pallidus). Ratings were given independently by the two experts and reconsidered in cases of large disagreement. The scores were then averaged together. The intra-class correlation coefficient (ICC) showed that the degree of the inter-rater agreement between these the two raters was very high (ICC = 0.82).

## Results

Overall, the sample included seven VS/UWS, four MCS−, one MCS+, and three eMCS patients. Due to excessive movement during acquisition, subject VS/UWS1 was excluded from the analysis (both AV and LS tasks), while the dataset of subjects VS/UWS3 and VS/UWS5 was partially excluded (AV and LS tasks, respectively).

### Whole-brain fMRI results in healthy participants

#### AV task

The group-based analysis on healthy participants allowed to define the brain areas involved during observation and listening of goal-related actions or intransitive movements performed with the mouth (AV task). The main results obtained with a univariate whole-brain approach are shown in Supplementary material (group results in Supplementary Fig. 2 and single-subject activation maps in Supplementary Figs 3 and 4). The mean statistical coordinates of cluster peak activations are reported in Supplementary Table 2. At group level, activations during ‘AV goal-related’ were present in low-level visual and auditory areas, including primary visual area (V1) and occipito-temporal cortex (MT/V5) and primary and secondary auditory areas in the superior temporal gyrus (STG). In addition, several high-level parietal and premotor areas showed increased signal change bilaterally, including the intraparietal sulcus (IPS) and the ventral premotor cortex (PMv). Other activated areas were the inferior frontal gyrus, pars opercularis and pars triangularis (IFGop, IFGtria), in the left hemisphere, and the supramarginal gyrus (SMG) in the right hemisphere. A very similar pattern was reported during ‘AV intransitive’, including activation of low-level primary visual and auditory areas. In addition, the high-level areas activated included both dorsal and ventral sectors of premotor cortex (PMd, PMv) and IFG (IFGop, IFGtria), more extended in the left hemisphere. Differently from ‘AV goal-related’, during ‘AV intransitive’, no activation was present in the IPS or SMG. During ‘AV ctrl’, the activated areas included mainly low-level visual and auditory areas, in the occipito-temporal cortex, bilaterally.

The direct contrast between ‘AV goal-related’ and ‘AV ctrl’ revealed the presence of activated clusters in both hemispheres, including IPS and IFGtria (Supplementary Fig. 2). These high-level areas include the main nodes of the MNS. Moreover, the direct contrast between ‘AV intransitive’ and ‘AV ctrl’ showed the presence of two clusters in the left hemisphere including dorsal and ventral premotor areas (PMv, PMd), IFGtria and IFGop.

#### LS task

The results related to the group-based analysis on the LS task in healthy participants are shown in Supplementary Fig. 2. In particular, listening to ‘action verbs’ was associated to increase signal change in the primary and secondary auditory areas, in the PMv and IFG, bilaterally. In addition, there was a left activation in the posterior parietal cortex (IPS). On the contrary, listening to ‘abstract verbs’ elicited mainly a bilateral activation of primary and secondary auditory areas in the temporal lobe.

The direct contrast between ‘LS action verbs’ and ‘LS abstract verbs’ revealed activations of IPS, PMv and IFG (both IFGop, IFGtria) only in the left hemisphere (Supplementary Fig. 2).

### Single-subject fMRI results in patients: whole-brain activations

#### AV task

**Figure 2:**
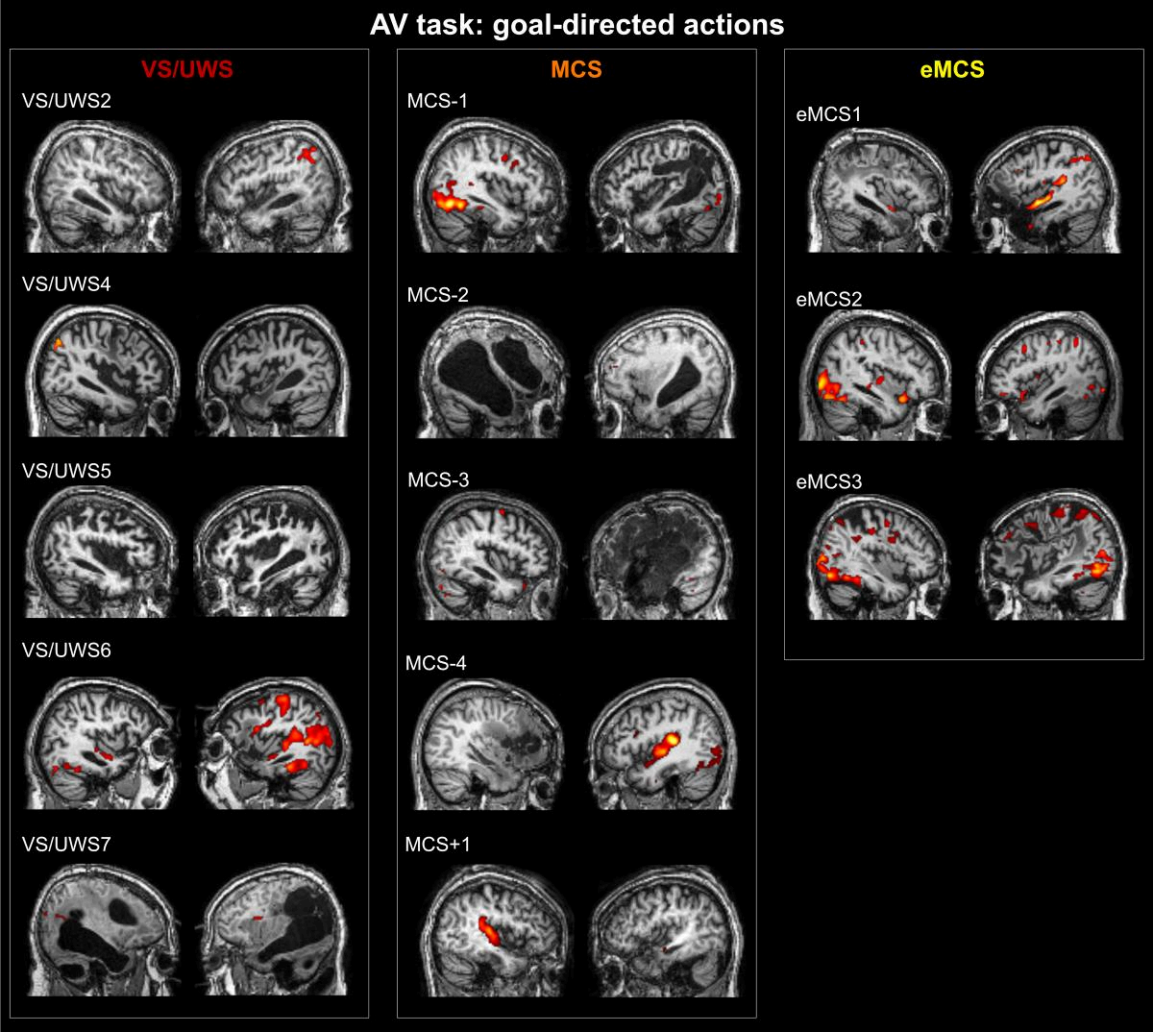
**Individual maps of brain activation of DOC patients (*n* = 15) during the AV task.** Functional maps are related to the contrast ‘AV goal-related’ versus ‘baseline’ (‘AV goal-related effect’). Significant activated voxels are rendered in two parasagittal sections of the corresponding anatomical normalized T1 volume. For all statistical maps, a voxel level threshold of *t* > 0.5, *P* < 0.001 uncorrected at voxel level and a cluster-level threshold (FDR corrected) of five voxels were applied.

The individual maps of brain activations found in DOC patients during ‘AV goal-related’ actions are shown in Fig. 2. Here and in the next figure, we mainly present this condition because it elicited more consistent activations among patients, as compared to ‘AV intransitive’ or ‘AV ctrl’. Note however that there was high variability between subjects concerning the condition (‘AV goal-related’, ‘AV intransitive’) able to produce significant activation. Based on the patients’ diagnosis, only one VS/UWS patient (VS/UWS6) showed an activation pattern comparable to that found in healthy participants, including activation of low-level auditory or visual areas, and other high order areas belonging to the parieto-premotor MNS. Among the remaining VS/UWS patients, two patients showed only a weak response in low-level visual and auditory areas (VS/UWS2 and VS/UWS7), while the other two patients did not show significant activations. Among the MCS−, two patients (MCS-1 and MCS-4) showed activation of high order areas, in particular of PMv, besides the activity in low-level visual/auditory areas. The remaining MCS− patients showed absent or very weak activations in low-level areas of one hemisphere. The MCS+ patient showed only activation of the primary auditory area in the right hemisphere. The three eMCS patients showed more extended activations including not only primary visual and auditory areas but also some clusters in the premotor and the parietal cortex, very likely including the main nodes of the MNS.

#### LS task

**Figure 3:**
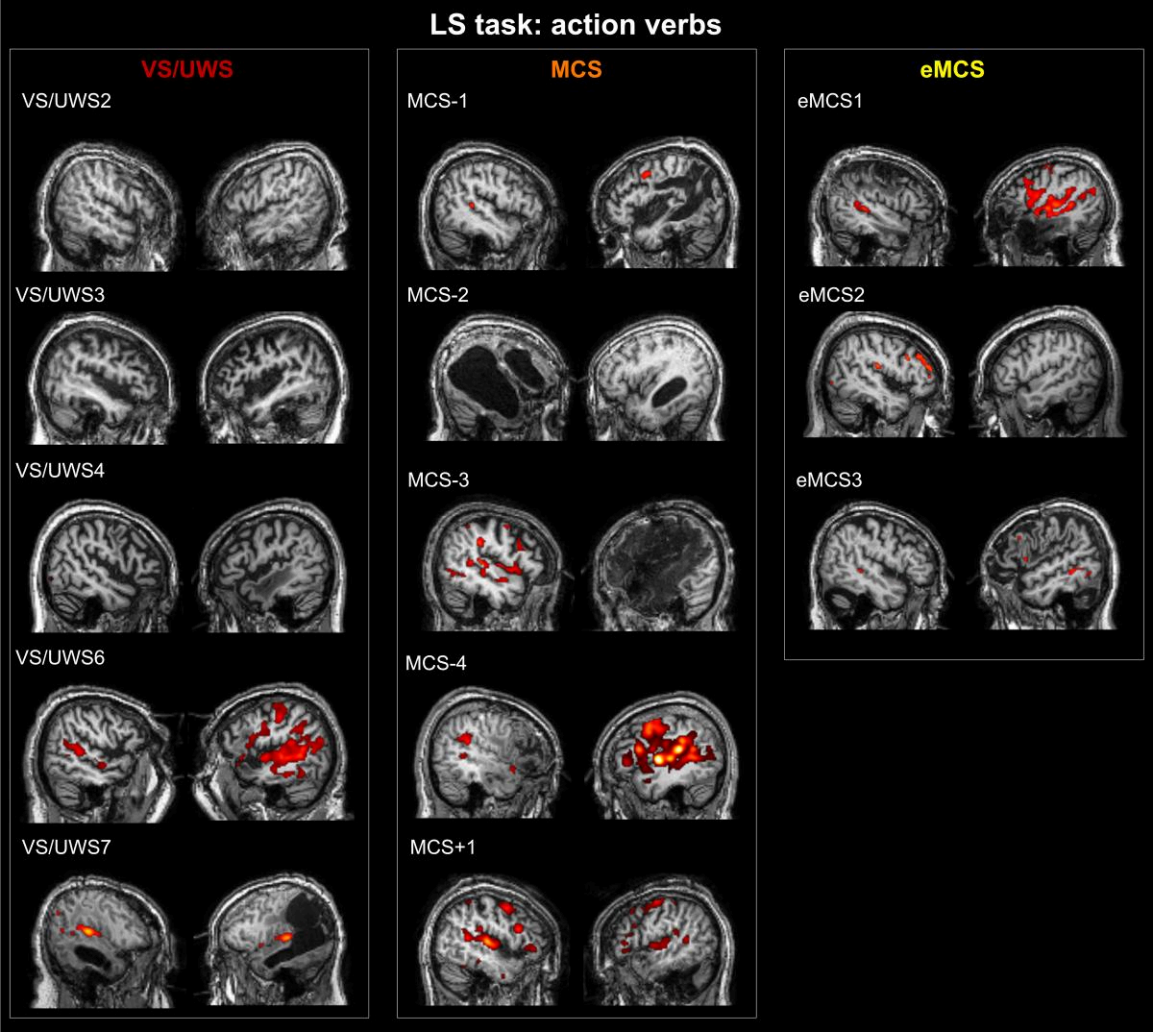
**Individual maps of brain activation of DOC patients (*n* = 15) during the LS task.** Functional maps are related to the contrast ‘LS action verbs’ versus ‘baseline’ (‘LS action verbs effect’). Activated voxels are rendered in two parasagittal sections of the corresponding anatomical normalized T1 volume. For all statistical maps, a voxel level threshold of *t* > 0.5, *P* < 0.001 uncorrected at voxel level and a cluster-level threshold (FDR corrected) of five voxels were applied.

During the LS task, the activations were more variable among patients and less strong, as compared to the AV task. Considering ‘LS action verbs’, only one patient (VS/UWS6) showed activation in high order parieto-premotor areas (Fig. 3). Note that the same patient showed a similar high order response also in the AV task. Another VS/UWS patient (VS/UWS7) showed only a low-level auditory response, consisting in a bilateral activation of the primary auditory area. Also, this result is congruent in the two tasks. The remaining VS/UWS patients did not show any significant activation. Concerning MCS− patients, two out of four showed cortical high order activations, including PMv, IFG (MCS-4) and the inferior parietal cortex (MCS-3). One patient (MCS-1) showed a weak activation at the level of the right primary auditory area, and the remaining patient did not show any significant activation (MCS-2). Interestingly, the MCS+ patient showed an extended bilateral pattern, which includes not only low-level but also high-level premotor and parietal areas in both hemispheres. Finally, all eMCS patients showed activations of primary auditory areas, and of high-level areas, in particular of PMv/IFG.

### ROI analysis results

#### Relation between ROIs activity and CRS-R scores

**Figure 4:**
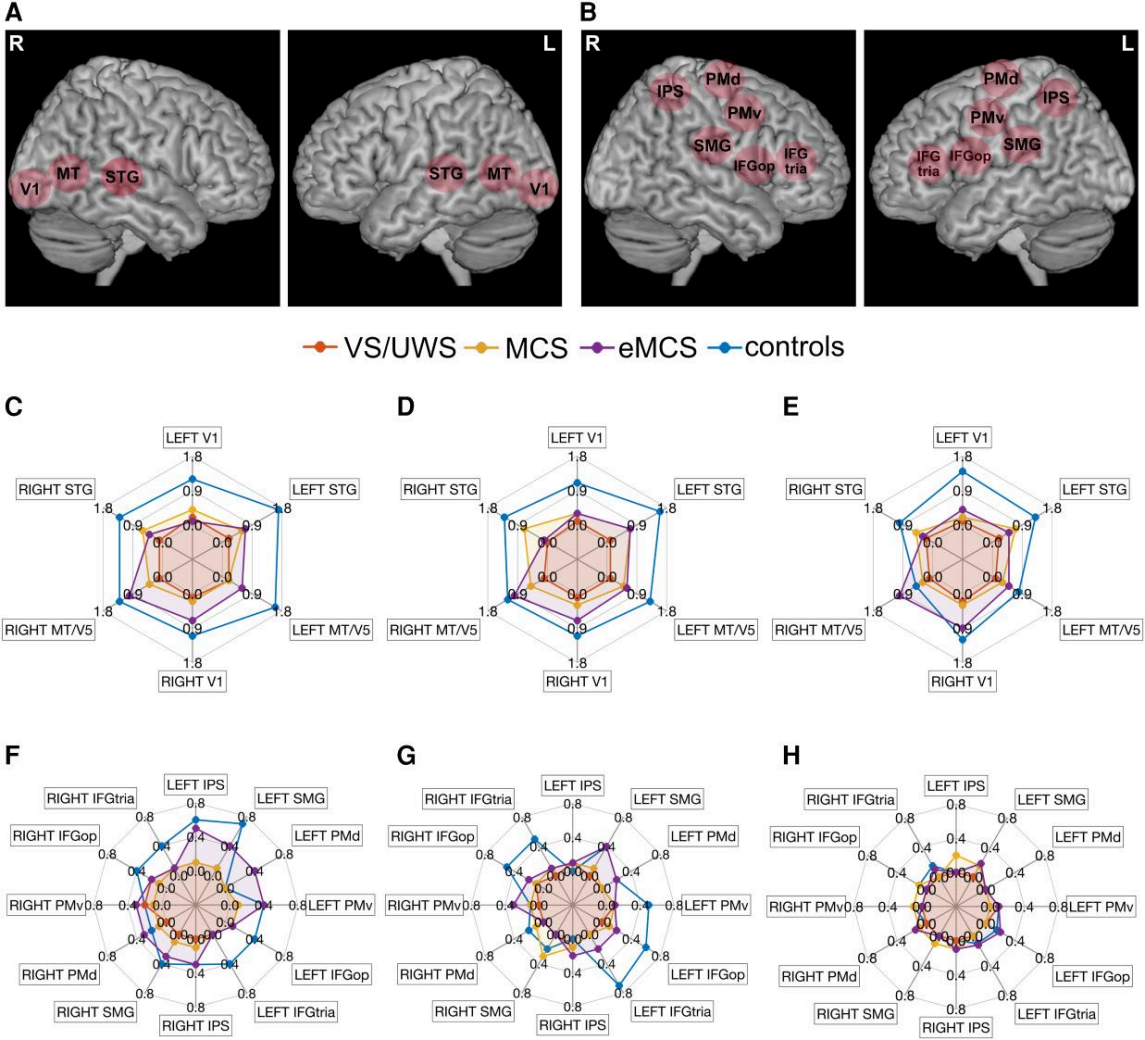
**Functional ROI analysis results (audiovisual task).** Activations during audiovisual (AV) task was investigated using a set of cortical ROIs, including low-level (**A**) and high-level cortical areas (**B**). The spider plots show averaged magnitude of activation (% signal change) in healthy control participants (*n* = 15), and DOC patients (*n* = 15), classified as eMCS (*n* = 3), MCS (*n* = 5) and VS/UWS (*n* = 7), respectively. Statistical threshold at voxel level was set at *t* > 0.5, *P* < 0.001 uncorrected at voxel level and a cluster-level threshold (FDR corrected) of five voxels were applied. The activation area corresponding to each AV condition is shown separately for low-level and high-level ROIs. (**C**) Activation area for ‘AV goal-related’ in low-level ROIs; (**D**) activation area for ‘AV intransitive’ in low-level ROIs; (**E**) activation area for ‘AV control’ (‘ctrl’) in low-level ROIs; (**F**) activation area for ‘AV goal-related’ in high-level ROIs; (**G**) activation area for ‘AV intransitive’ in high-level ROIs; (**H**) activation area for ‘AV control’ (‘ctrl’) in high-level ROIs. L, left hemisphere; R, right hemisphere.

**Figure 5:**
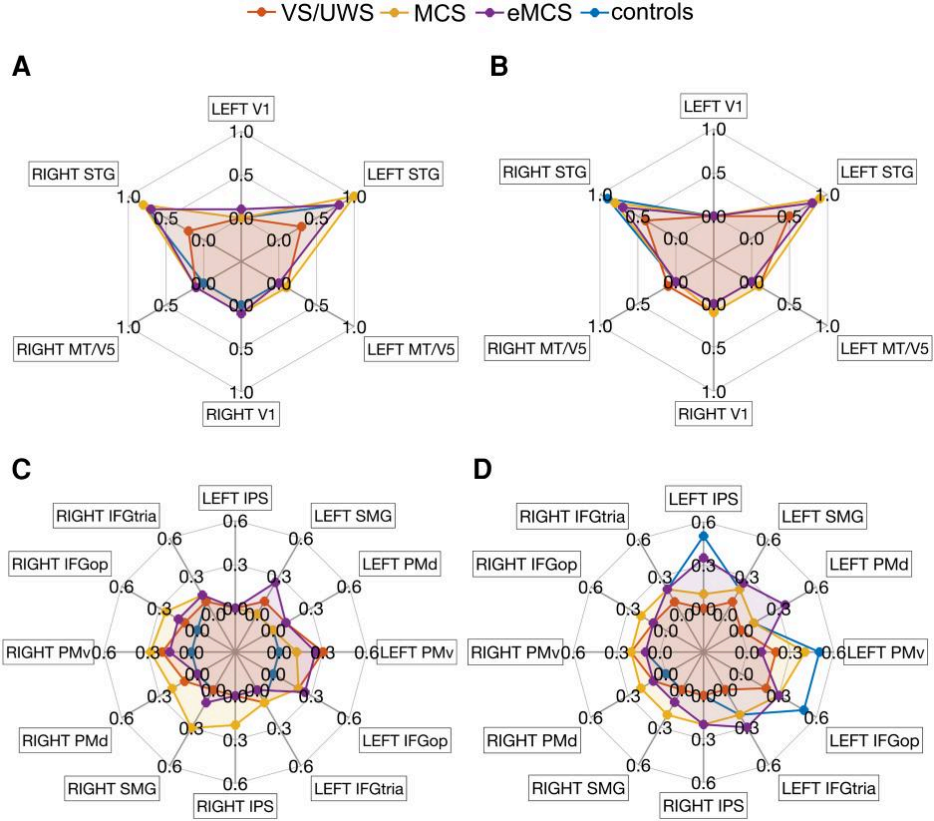
**Functional ROI analysis results (LS task).** Spider plots showing the averaged magnitude of activation (% signal change) during LS task, in healthy control participants (*n* = 15), and DOC patients (*n* = 15), classified as eMCS (*n* = 3), MCS (*n* = 5) and VS/UWS (*n* = 7), respectively. Statistical threshold at voxel level was set at *t* > 0.5, *P* < 0.001 uncorrected at voxel level and a cluster-level threshold (FDR corrected) of five voxels were applied. The activation area corresponds to each LS condition is shown separately for low-level and high-level ROIs. (**A**) Activation area for ‘LS action verbs’ in low-level ROIs; (**B**) activation area for ‘LS abstract verbs’ in low-level ROIs; (**C**) activation area for ‘LS action verbs’ in high-level ROIs; (**D**) activation area for ‘LS abstract verbs’ in high-level ROIs.

ROI analysis was carried out on selected regions belonging to the extended network activated in healthy participants during the AV and LS tasks. Figures 4 and 5 show the intensity of BOLD signal change for all the contrasts for AV and LS tasks, respectively, for healthy participants and patients grouped by type of diagnosis. Due to the low number of patients in each group, it is not possible to perform statistical differential analyses. Overall, it is possible to observe qualitatively that during the AV and LS tasks, the level of activation was lower in MCS and eMCS patients compared with healthy participants, and it progressively decreased passing from MCS to VS/UWS patients. The only difference concerns the level of activation in low-level auditory ROIs during the LS task (Fig. 5) in which the BOLD signal was comparable in MCS, eMCS patients and healthy participants, while it was significantly reduced in VS/UWS patients.

**Figure 6:**
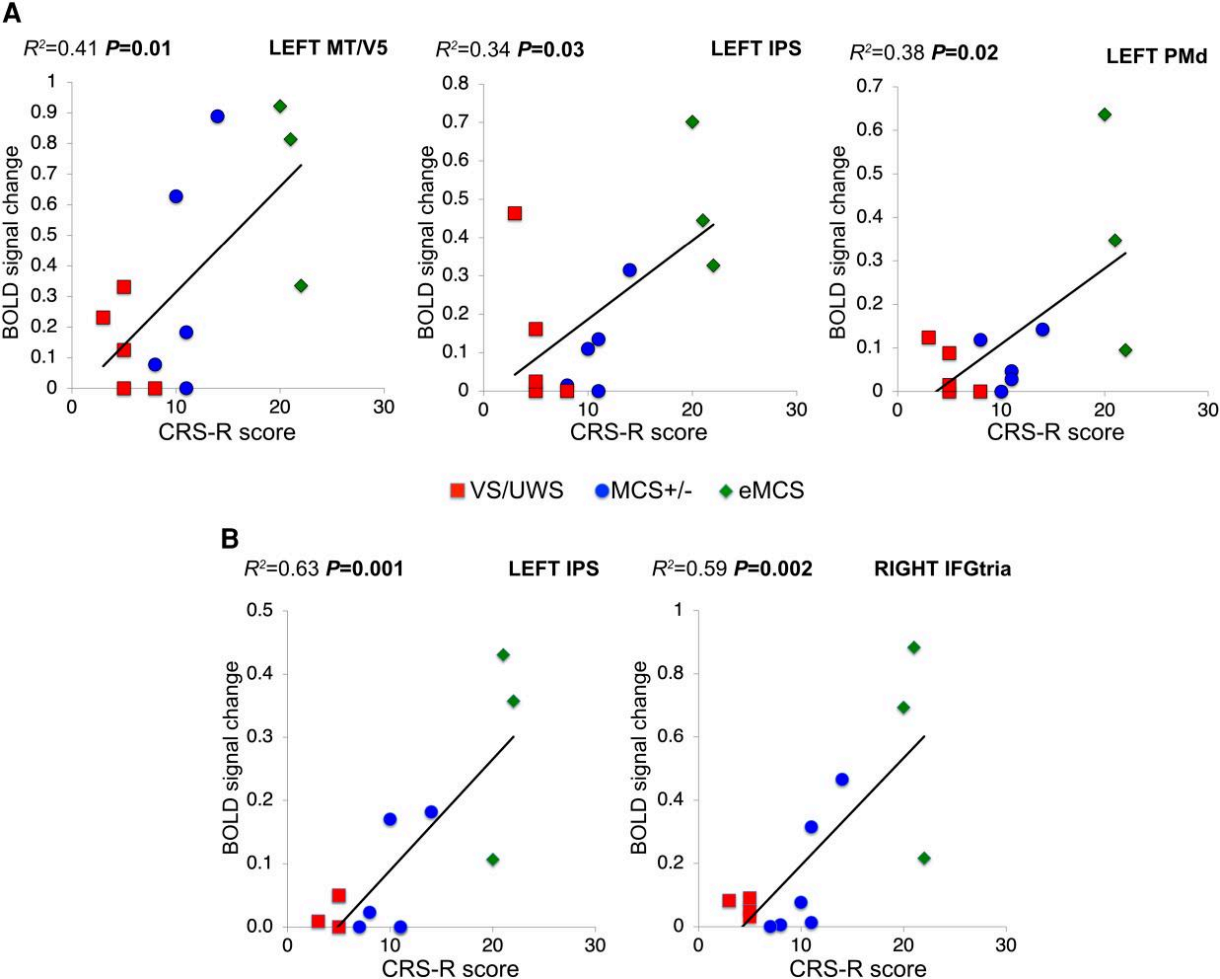
**Regression analysis results.** (**A**) Significant linear relation (*P* < 0.05) between brain activity (BOLD signal change) during AV task (‘AV goal-related’, ‘AV intransitive’) and the global CRS-R score in left MT/V5, left IPS and left PMd ROIs in DOC patients (*n* = 15). (**B**) Significant linear relation (*P* < 0.05) between brain activity (BOLD signal change) during LS task (‘LS action verbs’, ‘LS abstract verbs’) and the global CRS-R score in left IPS and right IFGtria in DOC patients (*n* = 15). BOLD, blood oxygen level dependent.

The results of the regression analysis (Fig. 6, Supplementary Table 3) showed a positive correlation between BOLD signal change during the AV task (contrasts: ‘AV goal-related effect’ and ‘AV intransitive related effect’) in left MT/V5, left IPS, left PMd and the CRS-R score. In addition, a positive correlation was also present between BOLD change during the LS task (contrasts: ‘LS action verbs effect’, ‘LS abstract verbs effect’) in left IPS, right IFGtria and the CRS-R score.

#### Patients’ classification based on ROIs functional activity

**Figure 7:**
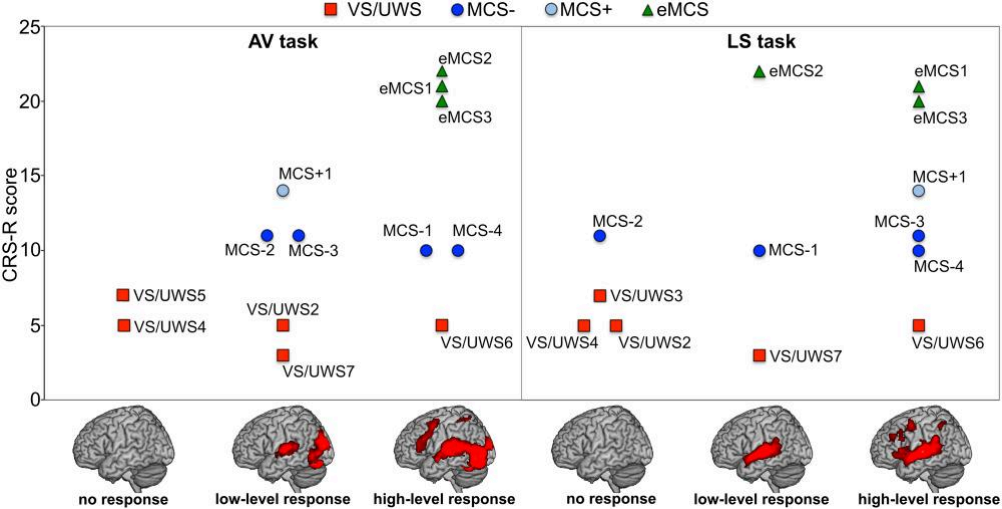
**Patients’ classification based on functional brain activity results.** Level of response showed by each DOC patient (*n* = 15, grouped by diagnosis) during the fMRI AV and LS tasks, plotted against their global CRS-R score. Three-dimensional lateral MNI renders at the bottom show left hemisphere activation resulting from the low-level and high-level audiovisual/auditory processing in healthy participants. The example of low-level response during AV task corresponds to the activation of primary visual and auditory areas, such as those observed during the processing of ‘AV ctrl’ naturalistic scenes, while the example of high-level response corresponds to the activation of parietal and premotor areas evoked by ‘AV goal-related’ actions and ‘AV intransitive’ movements. The functional response during the AV task was linearly related to the CRS-R score (*R*^2^ = 0.32, *P* = 0.02). Concerning LS task, the example of low-level response corresponds to the activation of primary auditory areas evoked by listening to ‘LS abstract verbs’, while high-level response shows the areas activated during ‘LS action verbs’. The activation level during LS task was moderately related to the CRS-R score, although not significant (*R*^2^ = 0.23, *P* = 0.06).

VS/UWS patients showed either no activation or residual activity in low-level and high-level areas, whereas all MCS patients presented functional response in low-level areas or in both low-level and high-level areas (Fig. 7). All eMCS patients showed activations in high-level areas, in at least one of the tasks. The linear regression analyses between the CRS-R score and the level of functional activation during tasks showed that the functional response during the AV task was linearly related to the CRS-R score (*R*^2^ = 0.32, *P* = 0.02), while the activation level during the LS task was moderately related to the CRS-R score, although not significant (*R*^2^ = 0.23, *P* = 0.06).

#### Structural alterations in DOC patients

Supplementary Fig. 5 shows structural alterations, within the considered ROIs, in patients grouped by diagnosis (VS/UWS, MCS, eMCS). Concerning the low-level auditory and visual ROIs, VS/UWS patients showed more severe damages, as indicated by lower scores (mean = 2.6 ± 0.2) as compared to MCS patients (mean = 2.9 ± 0.4) and eMCS patients (mean = 3.7 ± 0.3). In contrast, structural abnormalities in the high-level cortical ROIs and in the subcortical structures were comparable in all patients (Supplementary Table 4).

## Discussion

The present study assessed the presence of fMRI activity related to the MNS in patients with DOC and emerged from DOC, to provide a proof-of-principle for the applicability of action observation/listening as a therapeutic option in these patients. To this aim, participants underwent two passive tasks both known to elicit robust activation of the MNS in healthy individuals: one audiovisual task presenting short clips of goal-related or intransitive mouth actions, and one listening task administering mouth action verbs, accompanied by the respective control conditions.

The results show that: (i) many of the investigated patients with DOC and emerged from DOC have a residual activation of the MNS; while activations in low-level ROIs only were found in 5 out of 15 patients during AV task, and 3 out of 15 patients during LS task, activations in both low-level and high-level ROIs, including the MNS, were found in 6 out of 15 patients during both tasks; and (ii) although at group-level linear relation between the activity detected in some MNS ROIs and CRS-R scores were evident, at single-subject level the activity of this system appears to be dissociated from the level of consciousness, since high-level areas activations were observed not only in some patients with MCS and eMCS but also in one patient with a clinical diagnosis of VS/UWS.

###  

#### Activations during observation/listening of mouth actions

The activations found in healthy participants using the AV paradigm are consistent with those reported in previous studies on observation of mouth actions^24,27,56^ and listening to action sound.^30^ In particular, in the present study, goal-related actions activated bilaterally low-level visual and auditory areas in the occipito-temporal cortex, plus other sectors of the premotor and parietal cortex. AV intransitive actions activated a brain pattern similar to that described for goal-related actions, except the parietal cortex. During observation/listening of control stimuli, only low-level areas were recruited, confirming that premotor and parietal cortex (high-level areas) are crucial regions of the MNS system. These findings were evident not only at the group level but also in the activation maps of individual participants.

In patients, a range of patterns of activations was observed; some did not show any significant response to the AV stimuli, others, including patients clinically diagnosed as VS/UWS, showed significant responses, that were also anatomically appropriate and comparable with those of healthy individuals. These activations included mainly low-level visual and auditory areas, but also several sectors of the parietal and premotor cortex, mostly in MCS/eMCS patients but also evident in a VS/UWS patient. This would suggest a certain degree of processing of the observed/listened actions within the same motor circuits that in healthy individuals are involved in the execution of the same actions. Our findings are in line with previous fMRI works, reporting activations of low-level and high-level cortical areas in both VS/UWS and MCS patients, although these latter in a higher proportion compared to the former, during auditory, visual, somatosensory and olfactory stimulation.^57^

Moreover, our evidence is also in line with previous EEG investigations that show a specific desynchronization of the parietal and frontal regions in MCS patients during observation and imagination of hand/arm reaching-grasping movements.^41,42^ Although these effects were not reported at group level in VS/UWS, some of these patients with VS/UWS showed, as in our work, brain activity similar to healthy participants. Notably, we used a task consisting in the observation of mouth actions, associated with their corresponding sound. Considering the residual oro-facial movements present in DOC patients, we tried to induce motor resonance using the same motor repertoire.

#### Activations during listening to action/abstract verbs

Listening to both action and abstract verbs activates, in healthy participants, bilateral low-level auditory areas in the temporal cortex in line with other studies employing similar paradigms in healthy subjects.^58,59^ In addition, listening to action verbs recruited also sectors of premotor cortex and IFG, plus the IPS in the left hemisphere. These high-level activations are very likely associated with the processing of the action goal within the MNS.^31^

Some patients with a clinical diagnosis of MCS and one with VS/UWS showed cortical high-level activations, similar to those found during the AV task. This suggests, in some patients, the partial sparing of the auditory/linguistic channel, which would activate a motor resonance associated with listening to verbal stimuli. This finding corroborates previous evidence showing that automatic language processing can be observed in MCS, but also in VS/UWS patients,^47,60-62^ as indicated by the increase in activation within the linguistic networks.^3,60^

#### Relation between brain activation and clinical diagnosis

ROI analysis showed that the presence of residual response was generally stronger in eMCS than in MCS, and in MCS than in VS/UWS.^47,62,63^ In addition, a significant correlation was present between BOLD activation in temporal, premotor and parietal cortex (in particular MT/V5, IPS, PMd and IFG) and the level of clinical functioning assessed with CRS-R. Notably, these areas correspond to some of the main nodes of MNS described in humans. In fact, visual information elaborated within the temporal cortex (area MT/V5 and STS) is sent forward through the IPS to the PMv and the caudal part of the IFG.^64^ This pathway is known to provide information necessary for automatically understanding the observed motor act.^65^

#### Possible mechanisms producing activation of MNS in DOC patients

The structural alterations in DOC patients showed that low-level visual and auditory areas were mostly damaged in patients with diagnosis of VS/UWS as compared with MCS/eMCS patients, in line with findings reported on a larger sample of DOC patients.^55^ On the contrary, patients had similar alterations of high-level cortical areas and, most importantly, of subcortical structures, including midbrain, basal ganglia and thalamus. This finding is relevant for the interpretation of the presence of spared activation of parietal and frontal areas belonging to the MNS.^55,66^ Since the macroscopical damage of the subcortical regions and high-level cortical areas was quite similar in the various subgroups of patients, the absence of brain activity in most patients with VS/UWS cannot be explained only by this latter factor. It is therefore plausible that a prerequisite for the activation of the MNS is a certain degree of preservation of low-level visual and auditory cortical areas.

The activations found in some MCS/eMCS patients could rely on the presence of spared cortico-cortical, cortico-striatal and thalamo-cortical connections. This supports the proposal of a ‘mesocircuit’ model, according to which alterations of consciousness in DOC patients are associated to a downregulation of the activity occurring in the subcortical circuits, that results in a limited and fluctuating responsiveness.^11^ Furthermore, it has been reported that restoration of functions within basal ganglia-thalamic circuitry is more strongly associated with activation of parieto-frontal networks in patients showing gradual transition from coma to VS/UWS, MCS, confusional state and, ultimately, full cognitive recovery.^67,68^

The partial integrity of subcortical structures and of cortical areas in some VS/UWS and in MCS/eMCS patients suggests that action observation/listening can in principle stimulate cortical circuits, and, therefore, decrease the effect of inhibition in basal ganglia-thalamo-cortical circuits. However, it is also possible that the activation of subcortical and parieto-premotor circuits is not sufficient to generate a conscious experience linked to the observed/listened actions, due to the necessity of the restoration of more complex dynamic interactions between multiple cortical and subcortical networks.

#### Implication for diagnosis and stimulation interventions in DOC patients

These findings highlight the therapeutic potential of tailored interventions targeting the MNS (i.e. observational/listening based strategies) in patients with DOC. While it is evident that achieving a ‘real’ AOT in patients with DOC is not feasible, as it needs not only visual/auditory stimulation but also a reproduction/imitation component, the simple use of action observation/listening in DOC patients holds the potential for generating various positive effects.

First, the use of action observation/listening could, as other stimulation programmes, be a driver for more extensive activation of brain regions involved in high-level processes and in control of autonomic functions and arousal. Similarly, in acute settings, action observation/listening could support residual cognitive functions in principle limiting or avoiding rapid degradation of brain function. Secondly, by specifically facilitating corticomotor excitability, action observation/listening would promote or recover voluntary movement patterns, enabling or enriching the interaction between the patient and the environment. This is particularly important in the context of the CMD condition. It is now widely recognized that certain individuals categorized as VS/UWS may exhibit behavioural unresponsiveness attributed to a failure in motor output^12^ and not related to the low level of consciousness. This phenomenon is now termed CMD, also referred to as covert consciousness, functional locked-in syndrome, or non-behavioural MCS.^4^ This condition becomes apparent only through advanced neuroimaging techniques, such as task-based fMRI and EEG. Recent estimates indicate a considerable prevalence (15–20%) of patients with DOC in a CMD state,^8^ highlighting that a substantial population of these individuals are in this unrecognized condition, either due to a lack of appropriate assessment in standard healthcare settings or due to the limitations of the advanced neuroimaging techniques used.

Our proposal is that action observation and listening could be potentially used as tool to address this important CMD, possibly mitigating the misdiagnosis related to motor impairment, since this approach could, at least in principle, facilitate the activation of latent motor patterns and could act as a driving force for the interaction with the environment. The evidence of differential activations induced by the two employed tasks also suggests the implementation of personalized interventions based on the best stimuli (video, listening etc.) able to produce motor resonance in specific patients.

In addition, it could be predicted that patients with spared activations within low-level and high-level areas should be those who mostly benefit from action observation/listening. Among our patients, mainly MCS and eMCS, one patient with a clinical diagnosis of VS/UWS showed activations during both fMRI tasks. This patient was only 9 months post-injury, thus it could be very interesting to verify whether patients with similar features, in a long follow-up after action observation/listening stimulation, could show some sort of improvement.

### Limitations

Possible limitations of our study include the low sample size and presence of movements during acquisition, which led to the exclusions of some patients. However, the number of excluded patients is in line with similar works in the literature.^47^ Another limitation of our study is related to the fact that we did not perform visual evoked potentials before conducting the fMRI examination, which therefore did not allow us to assess the integrity of the visual pathways. The limited time in which patients could be tested and the common characteristics of the two tasks (both tasks had auditory stimulation, whereas only the AV task had visual stimulation) led us to choose auditory-evoked potentials. This obviously may have had an uncontrolled effect on the activations of the MNS which although supramodal may in principle have responded differently to stimuli perceived as auditory only than to coherent audiovisual stimuli. Concerning the used tasks, the passive brain activation during action observation/listening cannot be conclusive evidence of conscious processing in DOC patients. Nevertheless, the use of multiple and personalized fMRI tasks can provide useful information about the preserved networks, which in turn can represent the neural substrate supporting the recovery of cognitive and motor functions.

## Conclusion

Our results showed that the activation of the MNS through well-calibrated audiovisual stimuli leveraging on the supposed main motor reserve of patients (mouth movements) can occur automatically, and possibly without the involvement of awareness, as shown by its activity also in VS/UWS patients. The fact that the MNS is active in several DOC patients indicates that the neurophysiological basis for using action observation/listening as a sensory stimulation programme does exist in this challenging condition. Considering its low cost, ease of application even in home settings and virtual absence of undesirable effects, action observation and listening might be seriously considered as an integral part of the treatment and rehabilitation of these patients also in acute settings.

## Supplementary Material

fcae045_Supplementary_Data

## Data Availability

The data that support the findings of this study are available from the corresponding author, upon reasonable request.
