# Supplementary material for: Brain activation during processing of mouth actions in patients with disorders of consciousness
Source: Brain Commun. 2024 Feb 15;6(2):fcae045. doi: 10.1093/braincomms/fcae045 (PMC10907975; doi:10.1093/braincomms/fcae045)
Supplement: fcae045_Supplementary_Data [file fcae045_supplementary_data.docx]

**Brain activation during processing of mouth actions in patients with Disorders of Consciousness**

**Supplementary Material**

**MRI Acquisition and setup**

Whole-brain fMRI runs were acquired using a gradient-echo echo-planar imaging pulse sequence (TR = 2000 ms, TE = 30 ms, flip angle = 70°, voxel size = 2.5 mm^3^, matrix size = 90 × 95, 50 slices with 10% gap acquired in ascending order, volume number = 200, 3 dummy volumes). As part of the MRI scan, high-resolution structural 3D T1-weighted (T1w) image (TR = 9.86 ms, TE = 4.59 ms, FOV = 240x240 mm, voxel size = 1 mm^3^, flip angle = 8°, 185 sagittal slices) and a coronal fluid-attenuated inversion recovery (FLAIR) image were acquired. Sedation was never performed. The head was restrained using foam pillows.

**fMRI data preprocessing**

Data processing was performed with SPM12 (Wellcome Department of Imaging Neuroscience, University College, London, UK; http://www.fil.ion.ucl.ac.uk/spm) running on MATLAB R2022a (The Mathworks, Inc.). Structural images were centered and reoriented with functional images with respect to the anterior-posterior commissure axis. For each participant, all volumes were slice-timing corrected, spatially realigned to the first volume of the first functional run, and un-warped to correct for between-scan motion. Motion parameters were used as predictors of no-interest in the subsequent statistical analysis (see below) to account for translation and rotation along the three possible dimensions as determined during the realignment procedure. The cut-off used for motion correction tolerance was the size of the voxel (2mm). If motion exceeded this measure in translation and/or rotation, the dataset was not included in the analysis. T1-weighted image was segmented into grey, white and cerebrospinal fluid and spatially normalized to the Montreal Neurological Institute (MNI) space. Spatial transformation derived from this segmentation was then applied to the realigned EPIs for normalization and re-sampled in 2×2×2 mm voxels using trilinear interpolation in space. All functional volumes were then spatially smoothed with 8-mm full-width half-maximum isotropic Gaussian kernel (FWHM).

**
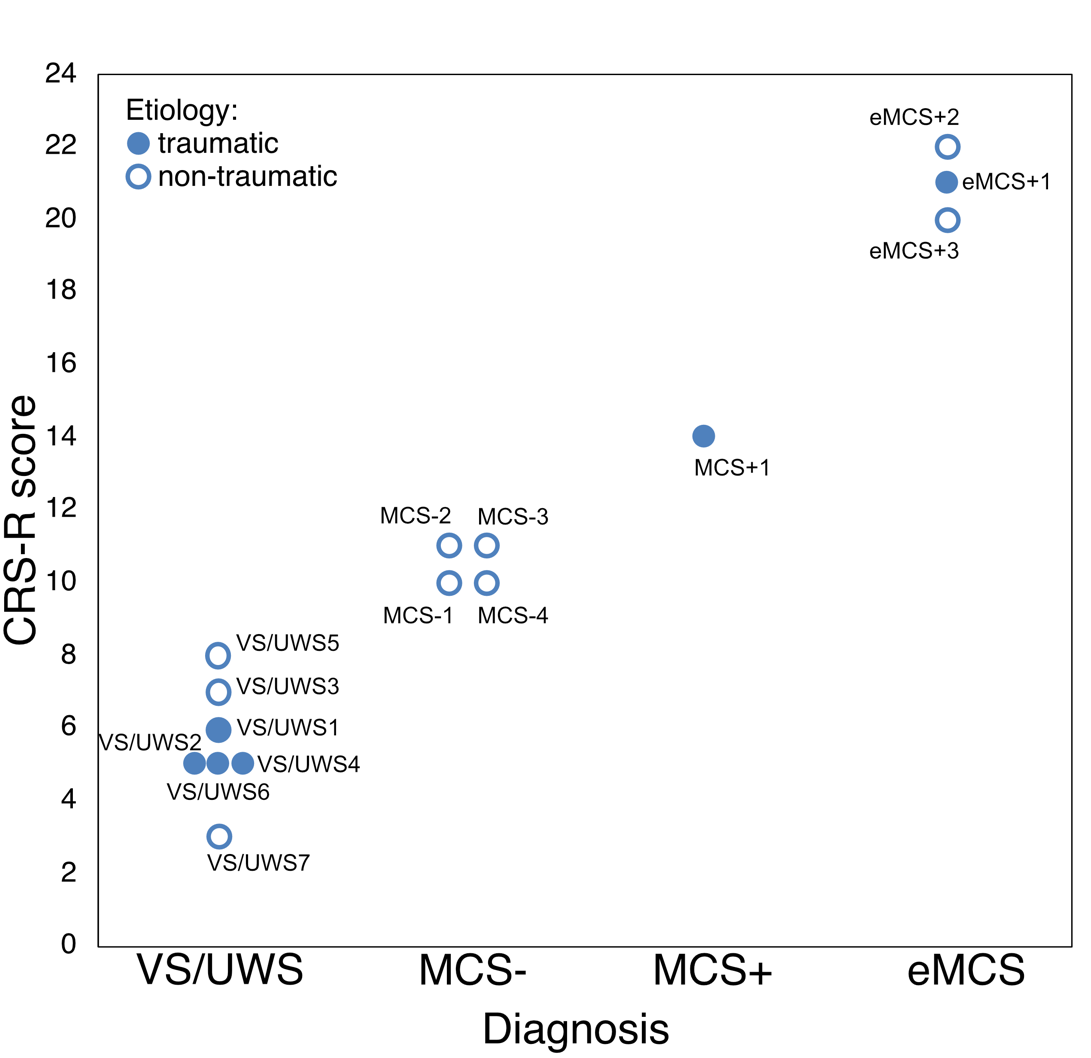
**

**Supplementary Fig. 1: Patients’ distribution according to clinical diagnosis, CRS-R score, and etiology.** Abbreviations: CRS-R, Coma Recovery Scale – revised.

**
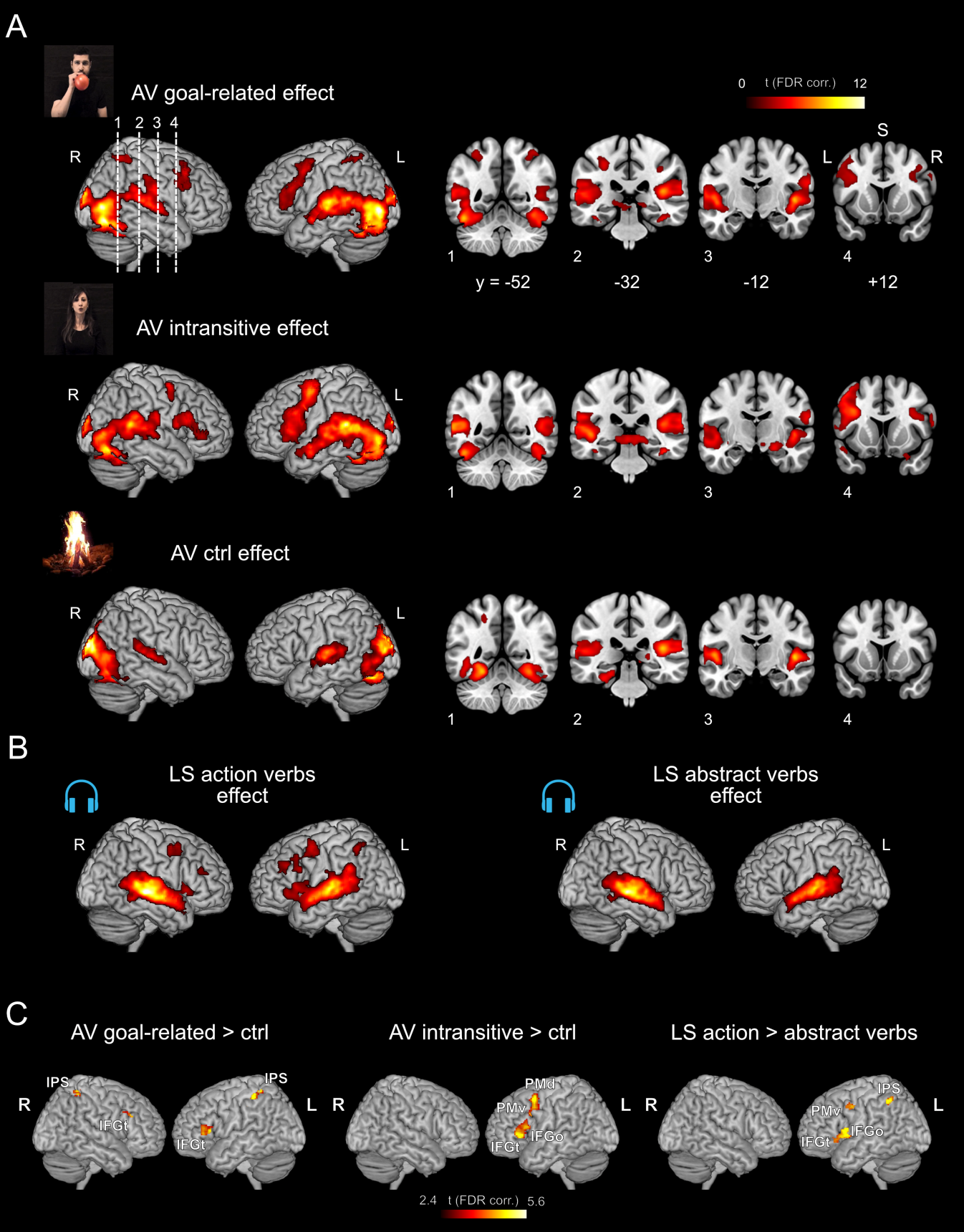
**

**Supplementary Fig. 2:** **Whole brain fMRI results in healthy participants**. **(A)** Brain activations of healthy participants during the AV task, related to the contrasts *AV goal-related* > *baseline*, *AV intransitive* > *baseline*, *AV ctrl* > *baseline*. **(B)** Statistical parametric maps during the LS task, related to the contrast *LS action verbs* > *baseline*, *LS abstract verbs* > *baseline*. **(C)** Direct contrasts between *AV goal-related* > *ctrl*, *AV intransitive* > *ctrl*, and *LS action* > *abstract verbs*. Activated voxels are rendered into a 3D MNI brain template (ch2better, lateral view, MRIcron software). The activation corresponding to the AV task are rendered also in 4 representative coronal slices. Activation maps are statistically significant with threshold set at *t*>0.5, *P*<0.001 (FDR corrected at cluster level), and minimum cluster size of 5 voxels. L = left hemisphere, R = right hemisphere.

**
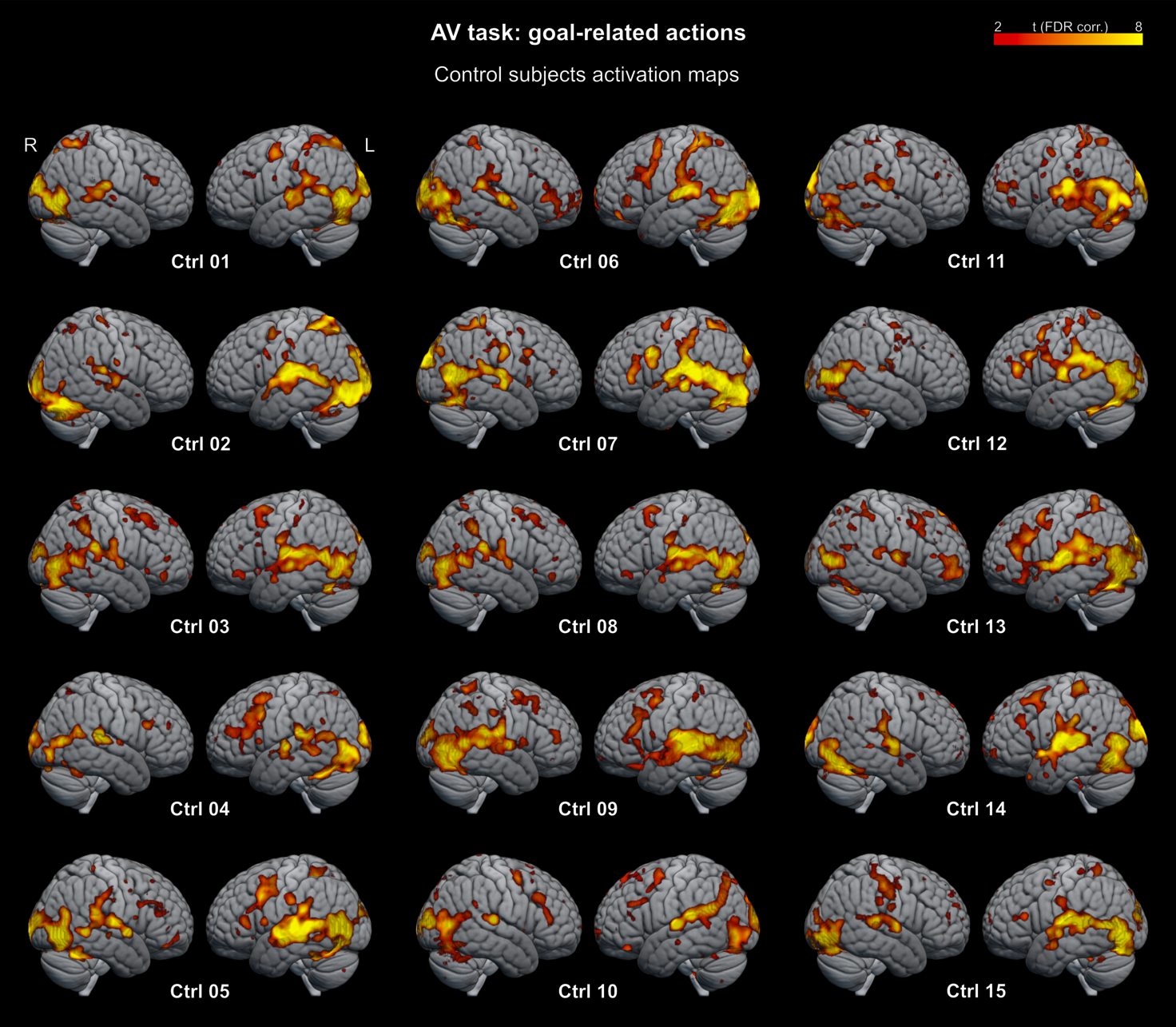
**

**Supplementary Fig. 3: Individual maps of brain activation of healthy controls during the AV task.** Functional maps are related to the contrast *AV goal-related* vs *baseline* (*AV goal-related effect*). Activated voxels are rendered into a 3D MNI brain template (ch2better, lateral view, MRIcron software). For all statistical maps a voxel level threshold of *t*>2, *P*<0.001 uncorrected at voxel level and a cluster-level threshold (FDR corrected) of 5 voxels were applied.

**
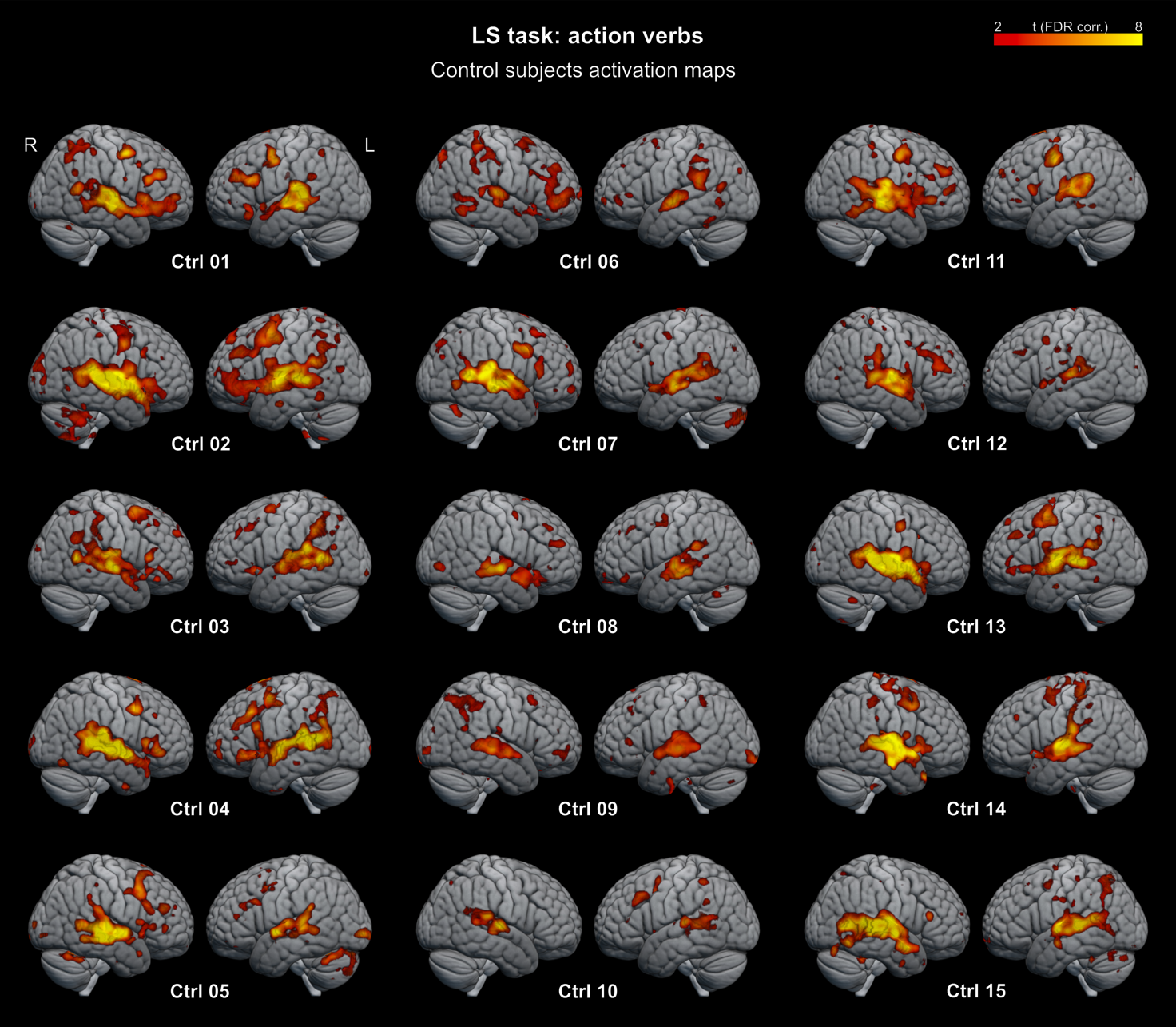
**

**Supplementary Fig. 4: Individual maps of brain activation of healthy controls during the LS task.** Functional maps are related to the contrast *LS action verbs* vs *baseline* (*LS action verbs effect*). Activated voxels are rendered into a 3D MNI brain template (ch2better, lateral view, MRIcron software). For all statistical maps a voxel level threshold of *t*>2, *P*<0.001 uncorrected at voxel level and a cluster-level threshold (FDR corrected) of 5 voxels were applied.


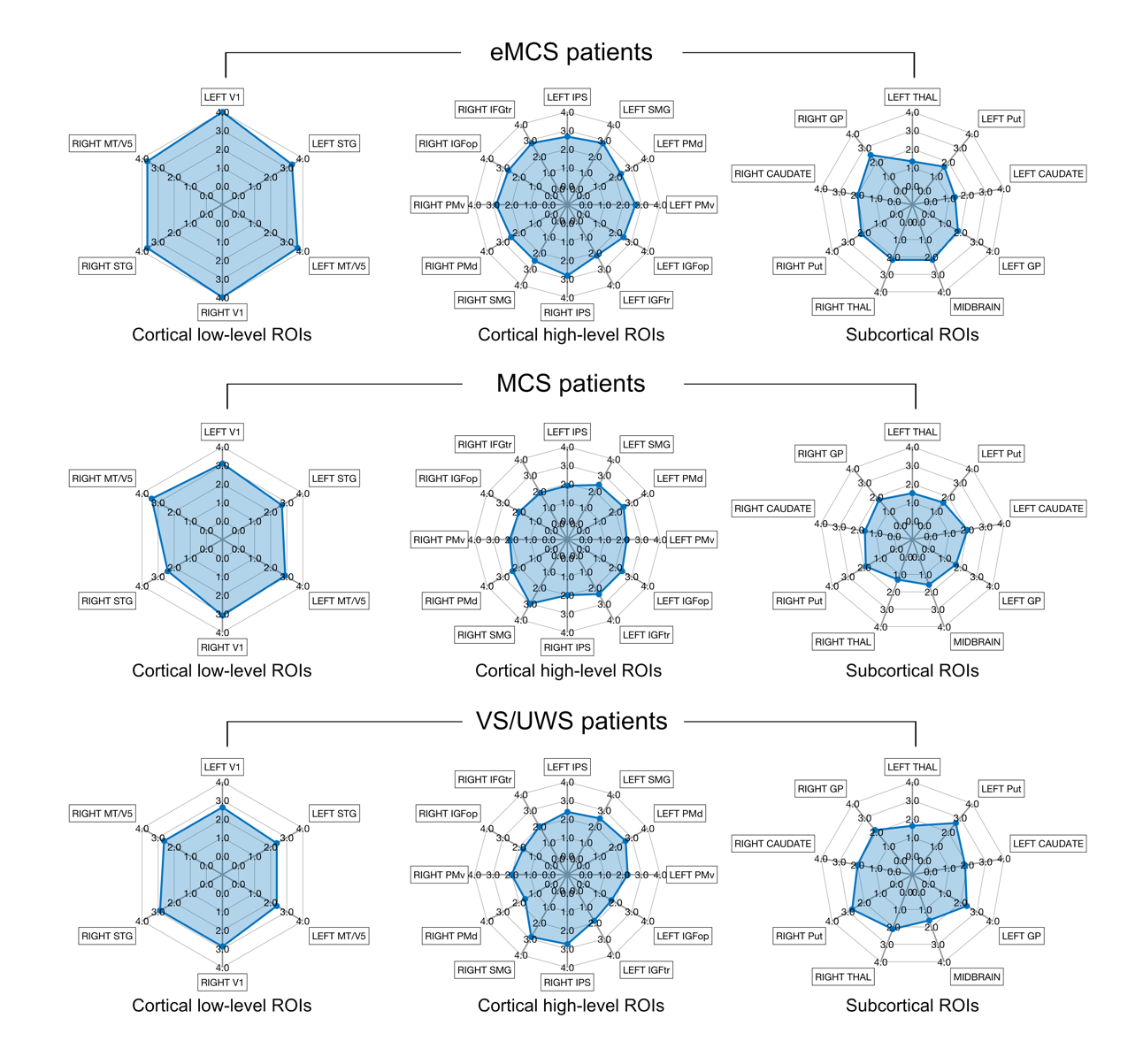


**Supplementary Fig. 5: Structural alterations in DOC patients.** Spider plots showing lesion distribution across cortical low level, cortical high level, and subcortical ROIs, in eMCS (N=3), MCS (N=5) and VS/UWS (N=7) respectively. Lower scores correspond to severe lesions. Score 0 = severely damaged; score 1 = recognizable but distorted morphology and/or severe signal abnormality; score 2 = moderate anatomical damage and/or signal abnormality; score 3 = mild anatomical damage and/or signal abnormality; score 4 = normal appearance. Ratings were given independently by the 2 experts and reconsidered in cases of large disagreement. The scores were then averaged together. Intra-class correlation coefficient (ICC) showed that the degree of the inter-rater agreement between these the two raters was very high (ICC=0.82).

**Supplementary Table 1 Mean, minimum and maximum, and *P* values of the considered variables for abstract and action verbs.**

| **Stimulus type** | **Phones** | **Syllables** | **Phonological neighbors** | **Sum token F** | **spoken FR** | **FAM** | **Valence** | **Arousal** | **IMG** | **CNC** |
| --- | --- | --- | --- | --- | --- | --- | --- | --- | --- | --- |
| **Action verbs** | 5.5 (4-7) | 2.25 (2-3) | 4.75 (1-11) | 108820.06 (33984-162288) | 3.75 (1-9) | 6.3 (5.7-6.9) | 4.35 (1.4-7) | 6.4 (4-7.1) | 6.92 (6.8-7) | 6.72 (6.3-7) |
| **Abstract verbs** | 5.75 (5-6) | 2.75 (2-3) | 5.75 (5-8) | 124288.37 (66289.5-168525) | 4.25 (2-7) | 6.07 (5.4-6.5) | 4.75 (3-7.1) | 6.35 (4.1-8.7) | 4.12 (3-4.9) | 2.3 (2.1-2.5) |
| ***P* value** | 0.886 | 0.343 | 0.343 | 0.486 | 0.686 | 0.486 | 0.886 | 0.686 | **0.029*** | **0.029*** |

FR=frequency; FAM= familiarity; IMG=imageability; CNC=concreteness.

**Supplementary Table 2. MNI coordinates and statistical details related to the brain functional activations in healthy participants.**

|  | ***left hemisphere*** | | | | ***right hemisphere*** | | | | |
| --- | --- | --- | --- | --- | --- | --- | --- | --- | --- |
|  | *z-score* | *peak MNI coordinates* | | | *z-score* | *peak MNI coordinates* | | | |
| **Anatomical Area** |  | x | y | z |  | x | y | z |  |
| ***AV goal-related effect*** |  |  |  |  |  |  |  |  |  |
| Primary visual area (V1) | Inf | -12 | -94 | 12 | Inf | 16 | -94 | 10 |  |
| Area MT/V5 | Inf | -46 | -66 | 4 | Inf | 48 | -74 | 2 |  |
| Superior temporal gyrus (STG) | Inf | -60 | -20 | 10 | Inf | 50 | -24 | 6 |  |
| Supramarginal gyrus (SMG) |  | -52 | -30 | 20 |  | 46 | -34 | 20 |  |
| Intraparietal sulcus (IPS) | 5.47 | -30 | -40 | 48 | 4.82 | 30 | -48 | 50 |  |
| Precentral gyrus (area PMd) | 5.00 | -42 | 0 | 58 |  |  |  |  |  |
| Precentral gyrus (area PMv) | 4.90 | -38 | 8 | 28 | 4.01 | 38 | 10 | 38 |  |
| Inferior frontal gyrus opercularis (IFGop) | 4.69 | -42 | 22 | 20 | 3.10 | 42 | 14 | 22 |  |
| Inferior frontal gyrus triangularis (IFGtria) | 4.98 | -54 | 24 | 24 | 3.45 | 58 | 18 | 26 |  |
|  |  |  |  |  |  |  |  |  |  |
| ***AV intransitive effect*** |  |  |  |  |  |  |  |  |  |
| Primary visual area (V1) | Inf | -12 | -94 | 12 | Inf | 16 | -94 | 10 |  |
| Area MT/V5 | Inf | -40 | -64 | 4 | Inf | 42 | -64 | 8 |  |
| Superior temporal gyrus (STG) | Inf | -58 | -26 | 10 | Inf | 54 | -28 | 10 |  |
| Precentral gyrus (area PMd) | 6.56 | -42 | 6 | 54 | 5.66 | 40 | 0 | 60 |  |
| Precentral gyrus (area PMv) | 7.22 | -36 | 10 | 28 | 4.66 | 36 | 10 | 26 |  |
| Inferior frontal gyrus opercularis (IFGop) | 6.91 | -42 | 20 | 20 | 4.53 | 46 | 12 | 20 |  |
| Inferior frontal gyrus triangularis (IFGtria) | 6.42 | -54 | 24 | 24 | 5.30 | 58 | 22 | 18 |  |
|  |  |  |  |  |  |  |  |  |  |
| **AV ctrl effect** |  |  |  |  |  |  |  |  |  |
| Primary visual area (V1) | Inf | -14 | -92 | 12 | Inf | 14 | -92 | 16 |  |
| Area MT/V5 | Inf | -40 | -64 | 2 | Inf | 40 | -66 | 4 |  |
| Superior temporal gyrus (STG) | Inf | -60 | -20 | 10 | Inf | 58 | -28 | 10 |  |
|  |  |  |  |  |  |  |  |  |  |
| ***LS action verbs effect*** |  |  |  |  |  |  |  |  |  |
| Superior temporal gyrus (STG) | Inf | -58 | -18 | 2 | Inf | 62 | -26 | 2 |  |
| Intraparietal sulcus (IPS) | 3.86 | -44 | -50 | 50 |  |  |  |  |  |
| Precentral gyrus (area PMv) | 4.49 | -50 | 6 | 44 | 3.58 | 52 | 2 | 46 |  |
| Inferior frontal gyrus opercularis (IFGop) | 4.33 | -50 | 20 | 4 |  |  |  |  |  |
| Inferior frontal gyrus triangularis (IFGtria) | 3.79 | -46 | 30 | -2 |  |  |  |  |  |
|  |  |  |  |  |  |  |  |  |  |
| ***LS abstract verbs effect*** |  |  |  |  |  |  |  |  |  |
| Superior temporal gyrus (STG) | Inf | -60 | -22 | 10 | Inf | 62 | -18 | 10 |  |
|  |  |  |  |  |  |  |  |  |  |
| ***AV goal-related vs ctrl*** |  |  |  |  |  |  |  |  |  |
| Intraparietal sulcus (IPS) | 5.56 | -30 | -44 | 50 | 3.24 | 38 | -34 | 38 |  |
| Inferior frontal gyrus triangularis (IFGtria) | 3.92 | -50 | 32 | 6 | 4.64 | 50 | 26 | 24 |  |
|  |  |  |  |  |  |  |  |  |  |
| ***AV intransitive vs ctrl*** |  |  |  |  |  |  |  |  |  |
| Precentral gyrus (area PMd) | 5.59 | -44 | 6 | 52 |  |  |  |  |  |
| Precentral gyrus (area PMv) | 5.20 | -40 | 14 | 30 |  |  |  |  |  |
| Inferior frontal gyrus opercularis (IFGop) | 5.14 | -46 | 18 | 14 |  |  |  |  |  |
| Inferior frontal gyrus triangularis (IFGtria) | 6.13 | -52 | 28 | 2 |  |  |  |  |  |
|  |  |  |  |  |  |  |  |  |  |
| ***LS action vs abstract verbs*** |  |  |  |  |  |  |  |  |  |
| Intraparietal sulcus (IPS) | 3.96 | -46 | -48 | 48 |  |  |  |  |  |
| Precentral gyrus (area PMv) | 3.35 | -34 | 8 | 38 |  |  |  |  |  |
| Inferior frontal gyrus opercularis (IFGop) | 4.93 | -56 | 8 | 12 |  |  |  |  |  |
| Inferior frontal gyrus triangularis (IFGtria) | 4.12 | -48 | 16 | 4 |  |  |  |  |  |

**Supplementary Table 3.** Statistical details of the regression analysis carried out to look for a linear relationship between BOLD activation in cortical ROIs during the AV and LST tasks, and the global CRS score. Bold font indicates significant effects.

|  | AV task | | |  | LST task | | |
| --- | --- | --- | --- | --- | --- | --- | --- |
| *Low-level ROIs* | *R^2^* | *F* | *P* |  | *R^2^* | *F* | *P* |
| Left V1 | 0.02 | 0.25 | 0.62 |  | 0.06 | 0.82 | 0.38 |
| Left STG | 0.18 | 2.49 | 0.14 |  | 0.09 | 1.14 | 0.30 |
| **Left MT/V5** | **0.41** | **7.79** | **0.01*** |  | 0.06 | 0.71 | 0.41 |
| Right V1 | 0.23 | 3.13 | 0.09 |  | 0.01 | 0.03 | 0.99 |
| Right STG | 0.13 | 1.69 | 0.21 |  | 0.13 | 1.73 | 0.21 |
| Right MT/V5 | 0.22 | 3.17 | 0.10 |  | 0.01 | 0.01 | 0.92 |
| *High-level ROIs* |  |  |  |  |  |  |  |
| **Left IPS** | **0.34** | **5.90** | **0.03*** |  | **0.63** | **19.39** | **0.001*** |
| Left SMG | 0.24 | 3.52 | 0.08 |  | 0.26 | 4.02 | 0.07 |
| **Left PMd** | **0.38** | **7.02** | **0.02*** |  | 0.13 | 1.78 | 0.20 |
| Left PMv | 0.19 | 2.74 | 0.12 |  | 0.11 | 1.44 | 0.25 |
| Left IFGop | 0.16 | 2.11 | 0.17 |  | 0.16 | 2.17 | 0.16 |
| Left IFGtria | 0.07 | 0.35 | 0.35 |  | 0.08 | 1.05 | 0.32 |
| Right IPS | 0.29 | 4.61 | 0.06 |  | 0.04 | 0.53 | 0.48 |
| Right SMG | 0.07 | 0.82 | 0.32 |  | 0.15 | 2.01 | 0.18 |
| Right PMd | 0.12 | 1.45 | 0.20 |  | 0.01 | 0.04 | 0.84 |
| Right PMv | 0.26 | 3.58 | 0.06 |  | 0.17 | 2.40 | 0.14 |
| Right IFGop | 0.19 | 2.46 | 0.11 |  | 0.24 | 3.58 | 0.08 |
| **Right IFGtria** | 0.10 | 1.22 | 0.24 |  | **0.59** | **15.93** | **0.002*** |

**Supplementary Table 4 Averaged ratings of structural alterations in cortical and subcortical ROIs.** Scores, assigned by two expert raters**,** indicate the severity of gross anatomical and signal abnormality in low-level cortical ROIs, high-level cortical ROIs and subcortical structures in both hemispheres. Lesion scores for each group of ROIs are reported separately based on clinical diagnosis. Lower scores correspond to extended lesions. Values between brackets indicate Standard Deviation.

| Patients | low-level ROIs | high-level ROIs | subcortical |
| --- | --- | --- | --- |
| VS/UWS | 2.6 (0.2) | 2.2 (0.4) | 2.1 (0.4) |
| MCS | 2.9 (0.4) | 2.3 (0.4) | 1.7 (0.2) |
| eMCS | 3.7 (0.3) | 2.6 (0.2) | 1.9 (0.4) |
